# Oncogenic *PIK3CA* promotes cellular stemness in an allele dose-dependent manner

**DOI:** 10.1073/pnas.1821093116

**Published:** 2019-04-04

**Authors:** Ralitsa R. Madsen, Rachel G. Knox, Wayne Pearce, Saioa Lopez, Betania Mahler-Araujo, Nicholas McGranahan, Bart Vanhaesebroeck, Robert K. Semple

**Affiliations:** ^a^Metabolic Research Laboratories, Wellcome Trust–Medical Research Council Institute of Metabolic Science, University of Cambridge, Cambridge CB2 0QQ, United Kingdom;; ^b^National Institute for Health Research, Cambridge Biomedical Research Centre, Cambridge CB2 0QQ, United Kingdom;; ^c^Centre for Cardiovascular Science, Queen’s Medical Research Institute, University of Edinburgh, Edinburgh EH16 4TJ, United Kingdom;; ^d^University College London Cancer Institute, University College London, London WC1E 6DD, United Kingdom;; ^e^Cancer Research UK Lung Cancer Centre of Excellence, University College London Cancer Institute, University College London, London WC1E 6DD, United Kingdom;; ^f^Cancer Genome Evolution Research Group, University College London Cancer Institute, University College London, London WC1E 6DD, United Kingdom;; ^g^Histopathology Department, Cambridge University Hospitals National Health Service Foundation Trust, Cambridge CB2 0QQ, United Kingdom

**Keywords:** PI3K, cancer, genetics, pluripotent stem cells, PROS

## Abstract

The *PIK3CA* gene, which encodes the p110α catalytic subunit of PI3 kinase (PI3K), is mutationally activated in cancer and in overgrowth disorders known as *PIK3CA*-related overgrowth spectrum (PROS). To determine the consequences of genetic *PIK3CA* activation in a developmental context of relevance to both PROS and cancer, we engineered isogenic human induced pluripotent stem cells (iPSCs) with heterozygous or homozygous knockin of *PIK3CA*^*H1047R*^. While heterozygous iPSCs remained largely similar to wild-type cells, homozygosity for *PIK3CA*^*H1047R*^ caused widespread, cancer-like transcriptional remodeling, partial loss of epithelial morphology, up-regulation of stemness markers, and impaired differentiation to all three germ layers in vitro and in vivo. Genetic analysis of *PIK3CA*-associated cancers revealed that 64% had multiple oncogenic *PIK3CA* copies (39%) or additional PI3K signaling pathway-activating “hits” (25%). This contrasts with the prevailing view that *PIK3CA* mutations occur heterozygously in cancer. Our findings suggest that a PI3K activity threshold determines pathological consequences of oncogenic *PIK3CA* activation and provide insight into the specific role of this pathway in human pluripotent stem cells.

## Significance

The *PIK3CA*^*H1047R*^ mutation is a common cancer “driver” and also causes an array of benign but highly disfiguring overgrowth disorders. Human induced pluripotent stem cells engineered to express two copies of *PIK3CA*^*H1047R*^ undergo cancer-like transcriptional remodeling and lose their ability to exit the stem cell state. A single mutant copy of *PIK3CA*^*H1047R*^, as observed in noncancerous overgrowth, had minimal effect on the stem cells and was fully compatible with normal differentiation. Combined with the finding of multiple *PIK3CA* mutant copies in human cancers, this suggests that a signaling threshold determines the disease consequences of *PIK3CA*^*H1047R*^, one of the commonest human oncogenic mutations.

Class IA phosphoinositide 3-kinases (PI3Ks) are essential components of the intracellular signaling cascades triggered by multiple growth factors, especially those acting via cell membrane receptor tyrosine kinases. Prominent among these are the insulin and insulin-like growth factor receptors. PI3K signaling is coupled to downstream activation of AKT and mammalian target of rapamycin complex 1 (mTORC1), which play key roles in organismal growth and development (1–3).

Strongly kinase-activating mutations in *PIK3CA*, the gene encoding the catalytic p110α subunit of PI3K, are among the most frequently observed oncogenic events in a range of human tumors (4). Although widely referred to as cancer “drivers,” the same mutations have also been identified in nonmalignant, albeit often severe, overgrowth disorders (5). These disorders are caused by postzygotic mosaic *PIK3CA* mutations and are phenotypically diverse, reflecting different patterns of mutation distribution and likely also different strengths of PI3K activation.

The commonest *PIK3CA* “hot-spot” variant, H1047R, has been studied extensively in cancer models, both in cells and in vivo. Endogenous, heterozygous expression in mice seemingly only results in cancer development in combination with additional oncogenic drivers (6–11), while transgenic overexpression of this *PIK3CA* mutant does lead to early malignancy (12–17). In cultured cells, *PIK3CA*^*H1047R*^ overexpression, but not heterozygous expression from the endogenous locus, leads to cellular transformation (18, 19). In human tumors, *PIK3CA* mutations are not mutually exclusive with other oncogenic alterations within the PI3K pathway (20), suggesting that stronger pathway activation may be required for malignant progression. This is supported by the benign nature of the overgrowth in *PIK3CA*-related overgrowth spectrum (PROS) where *PIK3CA*^*H1047R*^ heterozygosity is not sufficient to cause cancer. Despite this circumstantial evidence of dose-dependent effects of genetic PI3K activation, this has not been examined directly owing to the paucity of isogenic experimental models with endogenous expression of a defined number of oncogenic variants.

Disorders such as PROS illustrate that understanding aberrant development may hold lessons for cancer (21). Malignant transformation of cells typically involves dedifferentiation, reactivation of developmental pathways, and phenotypic plasticity. *PIK3CA*^*H1047R*^ was recently linked to induction of multipotency and cellular dedifferentiation in two mouse models of breast cancer (8, 16). Overexpression of wild-type (WT) *PIK3CA* in the head and neck epithelium of a mouse model of oral carcinogenesis has also been associated with dedifferentiation and epithelial-to-mesenchymal transition, increased transforming growth factor β (TGFβ) signaling, and up-regulated expression of the pluripotency factors *Nanog* and *Pou5f1* (*Oct3/4*) (22). Despite the insights gained from these and other mouse models of oncogenic *PIK3CA*, efforts to establish in vivo models of PROS have highlighted that species differences may constrain extrapolation from model organisms to the mechanisms of pathological PI3K activation in human disease (5).

Due to their unlimited self-renewal and differentiation capacity, human pluripotent stem cells (hPSCs) are increasingly used as tools to develop more relevant human disease models (23). Their inherent similarities to cancer cells also make them an attractive system in which to study oncogenic processes (24). Thus, to study dose-dependent effects of pathological PI3K hyperactivation in a developmental system of relevance to cancer and PROS, we engineered isogenic human induced pluripotent stem cells (iPSCs) to express *PIK3CA*^*H1047R*^ from one or both endogenous loci. Our data reveal clear dose-dependent developmental phenotypes downstream of p110α activation, with homozygosity but not heterozygosity for *PIK3CA*^*H1047R*^ promoting self-sustained stemness in vitro and in vivo. These findings emphasize the importance of using precisely engineered models of cancer-associated *PIK3CA* variants to obtain a faithful representation of their biological effects and have implications for our understanding of PI3K activation in human disease.

## Results

### Generation of Human iPSCs with Endogenous Expression of *PIK3CA*^*H1047R*^.

To establish a cell model suitable for interrogation of allele dose-dependent consequences of p110α activation in human development and disease, we used CRISPR/Cas9 genome editing of well-characterized, karyotypically normal WT iPSCs to generate multiple isogenic clones either heterozygous (*n* = 3) or homozygous (*n* = 10) for the activating *PIK3CA*^*H1047R*^ allele (*SI Appendix*, Fig. S1 *A*–*C*). To control for nonspecific effects caused by genetic drift following so-called bottleneck selection (25, 26), we expanded six WT clones exposed to the gene-targeting process. Sequencing of multiple clones of each genotype showed no evidence of mutagenesis of 17 computationally predicted CRISPR off-target sites (*SI Appendix*, Fig. S1*D*), and a normal karyotype was confirmed in three homozygous and two heterozygous clones more than 10 passages after targeting (*SI Appendix*, Fig. S1*E*).

**Fig. 1.**
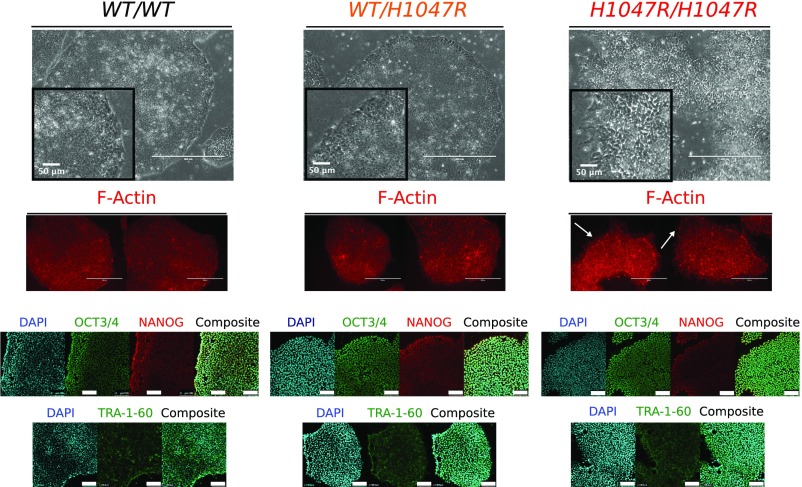
Isogenic hPSCs expressing *PIK3CA*^*H1047R*^ from one or both endogenous alleles. Representative light microscopy and immunofluorescence images of stem cell colonies from cultures with the indicated genotypes. F-Actin staining was used to visualize cell shape, and arrows highlight altered edge morphology and F-Actin–rich protrusions in *PIK3CA*^*H1047R/H1047R*^ colonies. (Scale bars: 400 µm; *Insets*, 50 µm.) Light micrograph images are representative of multiple clones from each genotype (4 WT, 3 *PIK3CA*^*WT/H1047R*^, and 10 *PIK3CA*^*H1047R/H1047R*^). The confocal images are of WT and mutant cells stained with antibodies against OCT3/4, NANOG, and TRA-1-60. Images are representative of at least two independent experiments and clones per genotype. (Scale bar: 100 µm.) See also *SI Appendix*, Fig. S1.

WT and *PIK3CA*^*WT/H1047R*^ colonies had a similar microscopic appearance, whereas *PIK3CA*^*H1047R/H1047R*^ clones exhibited aberrant colony morphology, characterized by disorganization of the normal epithelial appearance, including pronounced F-Actin-rich protrusions visible on colony margins (Fig. 1). Homozygous cells also proved more adherent in routine passaging, requiring longer dissociation time than WT and heterozygous cultures. Nevertheless, *PIK3CA*^*H1047R/H1047R*^ clones remained positive for the pluripotent stem cell markers NANOG, OCT3/4, and TRA-1-60 (Fig. 1), consistent with preserved stem cell identity.

### Allele Dose-Dependent Signaling Effects of *PIK3CA*^*H1047R*^.

**Fig. 2.**
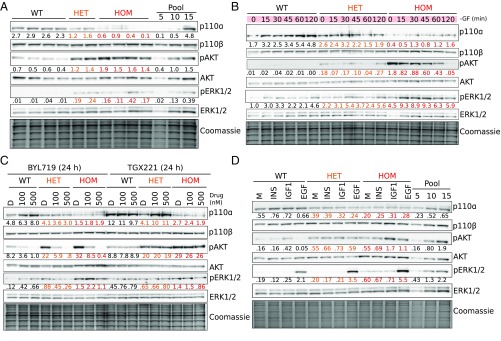
Graded activation of PI3K signaling in *PIK3CA*^*H1047R*^ hPSCs. Immunoblots are shown for p110α and p110β catalytic subunits of PI3K, and for total and phosphorylated AKT (S473) and ERK1/2 (T202/Y204; T185/Y187), with Coomassie-stained gels after transfer as loading control. Numbers below bands indicate quantification by densitometry (arbitrary units). (*A*) Signaling in cells collected 3 h after replenishment of growth factor (GF)-replete medium. Representative of at least three independent experiments. (*B*) Signaling time course during short-term GF depletion. Representative of at least two independent experiments. (*C*) Effects of 24 h of specific p110α or p110β inhibition in GF-replete medium using BYL719 or TGX221, respectively. DMSO (*D*) was used as control. Representative of two independent experiments. (*D*) Response of cells to 2 h of GF depletion followed by 20-min stimulation with 10 nM insulin (INS), insulin-like growth factor 1 (IGF1), or epidermal growth factor (EGF). GF-free DMEM/F12 medium (M) was used as control. The results are representative of two independent experiments. In all cases, independent clones of the same genotypes were used for replicate experiments. Protein pool dilutions are included where possible to assess assay performance (numbers represent micrograms). WT, wild type; HET, *PIK3CA*^*WT/H1047R*^; HOM, *PIK3CA*^*H1047R/H1047R*^. See also *SI Appendix*, Fig. S2.

We next assessed PI3K signaling in *PIK3CA*^*WT/H1047R*^ and *PIK3CA*^*H1047R/H1047R*^ iPSCs. p110α protein expression was reduced in both mutant genotypes and sometimes barely detectable in *PIK3CA*^*H1047R/H1047R*^ cells. Despite this, immunoblotting revealed graded increases in AKT phosphorylation across *PIK3CA*^*WT/H1047R*^ and *PIK3CA*^*H1047R/H1047R*^ lines, both in growth factor-replete conditions (Fig. 2*A*) and upon growth factor removal (Fig. 2*B*). Consistent with previous findings in breast epithelial cells heterozygous for *PIK3CA*^*H1047R*^ (19), both *PIK3CA*^*WT/H1047R*^ and *PIK3CA*^*H1047R/H1047R*^ cells also showed modest and graded increases in ERK phosphorylation.

Baseline PI3K pathway hyperactivation was inhibited in a dose-dependent manner by the p110α-specific inhibitor BYL719, while the p110β-specific inhibitor TGX221 had no effect (Fig. 2*C*). BYL719 did not reverse the allele dose-dependent down-regulation of the p110α protein, suggesting that it is not caused by acute negative-feedback mechanisms. In both mutant genotypes, low-dose BYL719 (100 nM) reduced AKT phosphorylation to the level in untreated WT cells (Fig. 2*C*), without inhibiting growth (*SI Appendix*, Fig. S2*A*). Relative to WT controls, mutant stem cells exhibited increased survival upon prolonged growth factor depletion, and this was also reversed by low-dose BYL719 (*SI Appendix*, Fig. S2*B*). A higher concentration of BYL719 (500 nM) was cytotoxic to both WT and *PIK3CA*^*WT/H1047R*^ cells (*SI Appendix*, Fig. S2*A*), but not *PIK3CA*^*H1047R/H1047R*^ cells, in which it reversed the aberrant colony morphology (*SI Appendix*, Fig. S2 *A* and *C*).

We also examined responses to acute stimulation with insulin, insulin-like growth factor 1 (IGF1), or epidermal growth factor (EGF) (Fig. 2*D*). *PIK3CA*^*WT/H1047R*^ and *PIK3CA*^*H1047R/H1047R*^ cells had high baseline AKT phosphorylation. This exceeded the level in IGF1-stimulated WT cells, but no consistent increase in the response to IGF1 was seen in mutant cells compared with WT (Fig. 2*D*). Insulin did not elicit discernible AKT phosphorylation in any of the iPSC cells used. This apparent insulin resistance may be caused by the high concentration of insulin (3 µM) used in the maintenance medium (27), resulting in down-regulation of insulin receptor expression at the plasma membrane (28). A modest increase in AKT phosphorylation in response to EGF was only observed in homozygous mutant cells. In contrast, EGF stimulation enhanced ERK phosphorylation above baseline in all iPSC lines, and this was progressively enhanced across heterozygous and homozygous mutant cells (Fig. 2*D*). These findings suggest that the MAPK/ERK pathway is primed to hyperrespond to growth factor stimulation in *PIK3CA*^*H1047R*^ stem cells, in an allele dose-dependent manner.

### Transcriptomic Effects of *PIK3CA*^H1047R^ in Pluripotent Stem Cells.

**Fig. 3.**
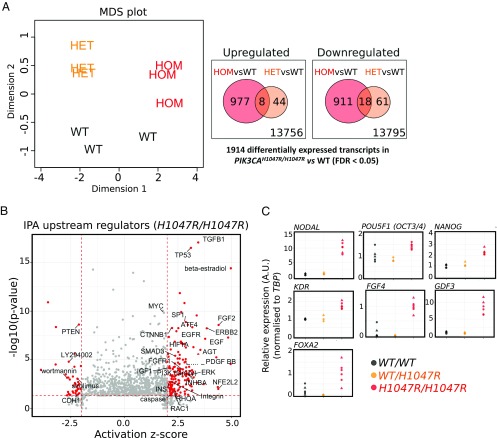
Widespread transcriptional remodeling in *PIK3CA*^*H1047R/H1047R*^ pluripotent stem cells. (*A*, *Left*) Multidimensional scaling (MDS) plot of transcriptomes of wild-type (WT), *PIK3CA*^*WT/H1047R*^ (HET), and *PIK3CA*^*H1047R/H1047R*^ (HOM) hPSCs profiled by RNA-seq. (*A*, *Right*) Venn diagrams showing overlap of up-regulated and down-regulated transcripts in *PIK3CA*^*H1047R*^ mutants compared with WT (FDR < 0.05, Benjamini–Hochberg; three clones per genotype). FC, fold change. (*B*) Ingenuity pathway analysis (IPA) of upstream regulators in *PIK3CA*^*H1047R/H1047R*^ cells, based on all differentially expressed genes. Components with absolute activation *z* score > 2 and *P* < 0.05 are highlighted in red. Selected components linked to PI3K signaling and pluripotency are labeled. (*C*) Assessment of expression of selected epiblast genes by real-time quantitative PCR, based on RNA-seq–specific pathway analyses. Data were obtained in two independent experiments. Expression values were scaled to the WT (*WT/WT*) or *PIK3CA*^*H1047R/H1047R*^ (*H1047R/H1047R*) mean as indicated. Individual points correspond to separate cultures: five WT (three clones), three *PIK3CA*^*WT/H1047R*^ (two clones), and six *PIK3CA*^*H1047R/H1047R*^ (four clones). All clones used for confirmation were distinct from those used to generate RNA-seq data. See also *SI Appendix*, Figs. S3 and S4.

To determine the wider dose-dependent consequences of genetic p110α activation, we profiled the protein-coding transcriptome of WT, *PIK3CA*^*WT/H1047R*^, and *PIK3CA*^*H1047R/H1047R*^ iPSCs, cultured in growth factor-replete conditions to mimic the in vivo milieu of the pluripotent epiblast. Multidimensional scaling demonstrated distinct transcriptomic signatures of WT, heterozygous, and homozygous cells (Fig. 3*A*). The transcriptome of *PIK3CA*^*WT/H1047R*^ cells was nearly identical to WT controls, with only 131 differentially expressed transcripts [false-discovery rate (FDR), 0.05]. In contrast, homozygosity for *PIK3CA*^*H1047R*^ led to differential expression of 1,914 genes (Fig. 3*A*). This indicates widespread transcriptional remodeling with a sharp allele dose dependency, suggestive of a threshold effect.

Kyoto Encyclopedia of Genes and Genomes annotation-based pathway analysis using all 1,914 differentially expressed genes in *PIK3CA*^*H1047R/H1047R*^ cells demonstrated significant changes to PI3K/AKT signaling, as expected. “Pathways in cancer” was identified as a common central node, highlighting the power of our isolated genetic activation of PI3K to recapitulate signatures identified in the genetically far more chaotic context of tumors (*SI Appendix*, Fig. S3). Other pathways identified as showing coherent perturbations were “Extracellular matrix-receptor interaction” and “Focal adhesion,” in keeping with the altered morphology and adhesion properties of homozygous mutants. Several genes involved in pluripotency regulation and WNT signaling were also differentially expressed. Finally, the TP53 pathway was found to be significantly altered (*SI Appendix*, Fig. S3). This is consistent with prior evidence of TP53 activation in cell lines with hyperactivation of PI3K/AKT (29–32). However, given the recent report that a substantial proportion of iPSC lines have *TP53* mutations (33), we sequenced the *TP53* gene of all clones. We found that two of the WT lines were indeed heterozygous for *TP53* C135F (*SI Appendix*, Fig. S4*A*), a mild loss-of-function allele based on biochemical assays in yeast (34). Despite this, inspection of each iPSC clone’s RNA-seq data for the differentially expressed TP53 signaling genes showed that the signature difference in *PIK3CA*^*H1047R/H1047R*^ cells was not attributable to these two WT lines.

To identify potential drivers of the transcriptional changes in *PIK3CA*^*H1047R/H1047R*^ cells, we also undertook Ingenuity pathway analysis of upstream regulators. This again revealed the expected activation of PI3K/AKT signaling. It also implicated factors important in stem cell regulation, including TGFβ, FGF2, TP53, β-catenin, and MYC (Fig. 3*B*). TGFβ was the most significant prediction, and supporting increased signaling within this pathway, we found increased phosphorylation of SMAD2 in homozygous mutants (*SI Appendix*, Fig. S4*B*). These cells also had up-regulated expression of *NODAL* (Fig. 3*C* and *SI Appendix*, Fig. S3), a member of the TGFβ superfamily that maintains the pluripotent epiblast at early developmental stages and later induces primitive streak formation during gastrulation (35). Consistent with NODAL’s dual function, *PIK3CA*^*H1047R/H1047R*^ cells exhibited a stemness signature (36) including up-regulation of *NANOG*, *POU5F1* (*OCT3/4*), *MYC*, *KDR*, *IGF1R*, as well as up-regulation of primitive streak markers such as *FGF4*, *GDF3*, and *FOXA2* (Fig. 3*C* and *SI Appendix*, Fig. S3). Up-regulation of *NODAL* in WT and mutant cells was abolished by p110α-specific inhibition with BYL719 (*SI Appendix*, Fig. S4*C*). In comparison, *NANOG* expression remained mostly unaffected by BYL719, with a trend toward down-regulation after 48 h of p110α inhibition (*SI Appendix*, Fig. S4*C*). These findings suggest up-regulation of *NODAL* and enhanced TGFβ/SMAD2 signaling as a candidate mechanism whereby p110α activation may exert effects on stemness in hPSCs.

### Homozygosity for *PIK3CA*^*H1047R*^ Confers Self-Sustained Stemness upon Embryoid Bodies.

**Fig. 4.**
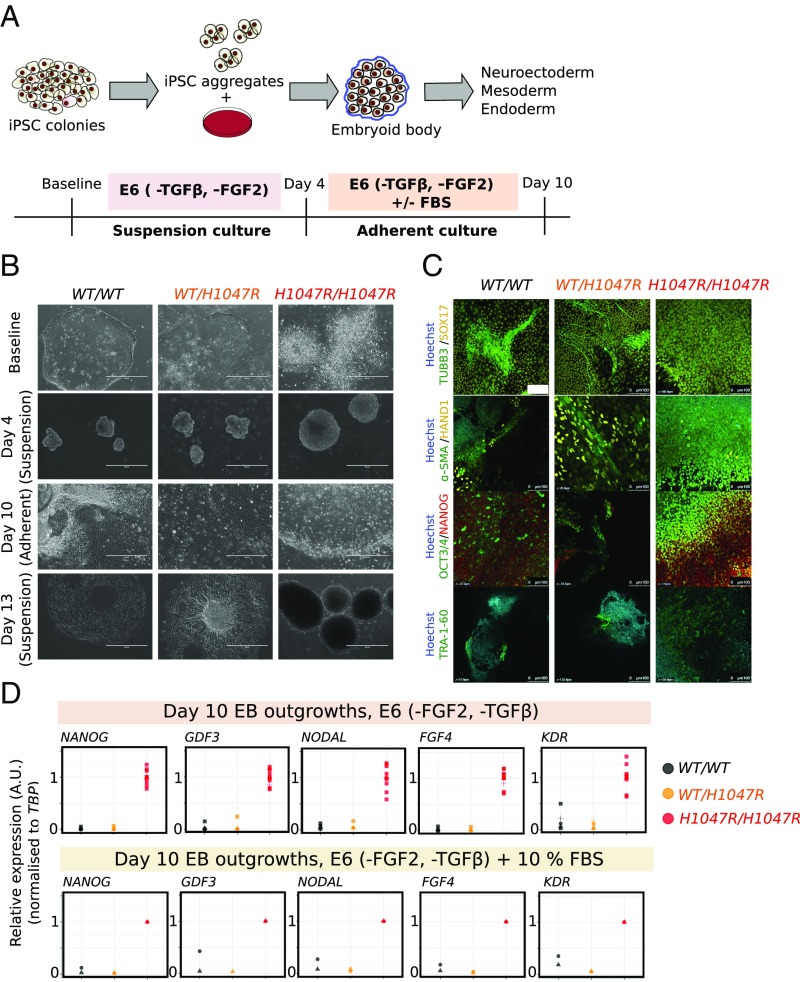
Self-sustained stemness in *PIK3CA*^*H1047R/H1047R*^ embryoid bodies (EBs). (*A*) Schematic illustrating the protocol used for EB formation and subsequent adherent culture. E6, Essential 6 medium; FGF2, fibroblast growth factor 2; TGFβ, transforming growth factor β. (*B*) Representative bright-field micrographs of WT (*WT/WT*), *PIK3CA*^*WT/H1047R*^ (*WT/H1047R*), and *PIK3CA*^*H1047R/H1047R*^ (*H1047R/H1047R*) cells at baseline (iPSC stage), 4 d (suspension), 10 d (adherent), and 13 d (suspension) following EB formation. *PIK3CA*^*H1047R/H1047R*^ iPSC colonies are refractile due to partial dissociation, while stem cell-like colonies emerging from adherent *PIK3CA*^*H1047R/H1047R*^ EBs are highly compact. In addition to the floating layers of differentiated cells shown here, WT and *PIK3CA*^*WT/H1047R*^ suspension cultures on day 13 also contained larger EB aggregates with complex morphologies and internal differentiation. (Scale bar: 400 μm.) (*C*) EB outgrowths were fixed on day 10 and stained for TRA-1-60 or costained for TUBB3/SOX17, α-SMA/HAND1 or NANOG/OCT3/4. Hoechst was used for nuclear visualization. Images are representative of two independent experiments, using a single WT clone and two clones of each mutant. (Scale bar: 100 µm.) (*D*) Real-time quantitative PCR analysis of stemness gene expression in EB outgrowths in E6 medium without TGFβ and FGF2. Individual replicates shown in the panel are from three to four WT clones, two *PIK3CA*^*WT/H1047R*^ clones, and four *PIK3CA*^*H1047R/H1047R*^ clones (including technical duplicates of the *PIK3CA*^*H1047R/H1047R*^ outgrowth cultures). Expression values are in arbitrary units (A.U.). See also *SI Appendix*, Fig. S5.

Embryoid bodies (EBs) are widely used to model lineage specification during gastrulation (37, 38). Previous studies have shown that *NODAL* overexpression in hPSC-derived EBs blocks differentiation to all three germ layers (39). Given the evidence for up-regulated *NODAL* and TGFβ signaling in *PIK3CA*^*H1047R/H1047R*^ cells, we tested whether the resulting EBs would behave similarly to NODAL-overexpressing EBs. EBs were established without TGFβ and FGF2, cultured in suspension for 4 d, and allowed to generate adherent outgrowths for 6 d (Fig. 4*A*). *PIK3CA*^*H1047R/H1047R*^ stem cells consistently generated compact, cystic EBs that failed to bud and undergo internal reorganization (Fig. 4*B*), with notable resemblance to mouse EBs overexpressing constitutively active PDK1 or AKT1 (40). In adherent culture, *PIK3CA*^*H1047R/H1047R*^ EB outgrowths resembled stem cell colonies (Fig. 4*B*). Confirming this, *PIK3CA*^*H1047R/H1047R*^ EB outgrowths stained positive for the stemness markers OCT3/4, NANOG, and TRA-1-60 (Fig. 4*C*). WT and *PIK3CA*^*WT/H1047R*^ EBs, in contrast, exhibited complex morphologies in suspension and yielded heterogeneous outgrowths of differentiated cells, which continued to mature during the experiment (Fig. 4 *B* and *C*).

The apparent differentiation block of *PIK3CA*^*H1047R/H1047R*^ EBs was assessed transcriptionally using lineage-specific arrays and candidate gene quantitative PCR. Unlike WT and *PIK3CA*^*WT/H1047R*^ EBs, homozygous mutants exhibited sustained expression of stemness genes and failed to up-regulate germ layer-specific markers, both in adherent cultures and in suspension (Fig. 4*D* and *SI Appendix*, Fig. S5 *A*–*D*). This phenotype persisted in the presence of serum, which is used to induce EB differentiation (Fig. 4*D* and *SI Appendix*, Fig. S5*A*). Attempts to reverse the *PIK3CA*^*H1047R/H1047R*^ EB phenotype with the p110α inhibitor BYL719 were unsuccessful due to poor EB survival in the presence of the drug, consistent with previous studies demonstrating high EB sensitivity to PI3K/mTOR inhibition (40–42).

### Heterozygosity for *PIK3CA*^*H1047R*^ Is Compatible with Directed Definitive Endoderm Formation.

**Fig. 5.**
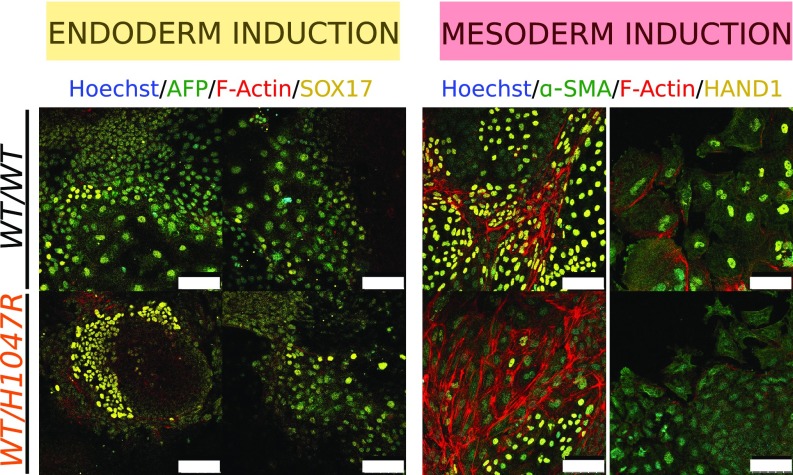
Heterozygosity for *PIK3CA*^*H1047R*^ does not affect endoderm or mesoderm differentiation in EBs. Representative confocal images of WT (*WT/WT*) and *PIK3CA*^*WT/H1047R*^ (*WT/H1047R*) outgrowths on day 10 of the differentiation protocol, stained with antibodies against endoderm (AFP/SOX17) and mesoderm (α-SMA/HAND1)-specific markers. Hoechst was used for nuclear visualization and F-Actin for cell boundary demarcation. The images are from one clone per genotype. (Scale bar: 100 μm.) See also *SI Appendix*, Fig. S6.

Heterozygosity for *PIK3CA*^*H1047R*^ did not produce major perturbations in the transcriptome of iPSCs nor in EB differentiation. Nevertheless, observation of *PIK3CA*-driven overgrowth in PROS suggests that mesodermal and neuroectodermal tissues are widely involved, while tissues of endodermal origin are only rarely affected by strong activating mutations, raising the possibility of negative selection during endodermal development (5). We thus sought to undertake more systematic analysis of early differentiation in our human developmental models of *PIK3CA*^*H1047R*^. To overcome the high variability seen in self-aggregating, spontaneously differentiating EBs, the protocol was modified, incorporating use of microwell plates to ensure homogeneous EB size (*SI Appendix*, Fig. S6*A*). EB formation was followed by 3 d of exposure to different concentrations of Activin A, BMP4, and FGF2 to promote mesoderm or definitive endoderm formation (43, 44). Lineage-specific gene expression arrays, candidate gene quantitative PCR, and immunostaining assays were used to assess expression of multiple differentiation markers. Mesoderm or endoderm induction led to increased expression of the expected lineage-specific markers (*SI Appendix*, Fig. S6 *B* and *C*). The temporal pattern and relative expression levels of the analyzed genes were similar for *PIK3CA*^*WT/H1047R*^ and WT EBs (*SI Appendix*, Fig. S6 *B* and *C*), and adherent outgrowths from both stained positive for mesoderm and endoderm markers at the end of the 10-d protocol (Fig. 5). The results of this assay argue against an inability of *PIK3CA*^*WT/H1047R*^ iPSCs to yield definitive endoderm.

**Fig. 6.**
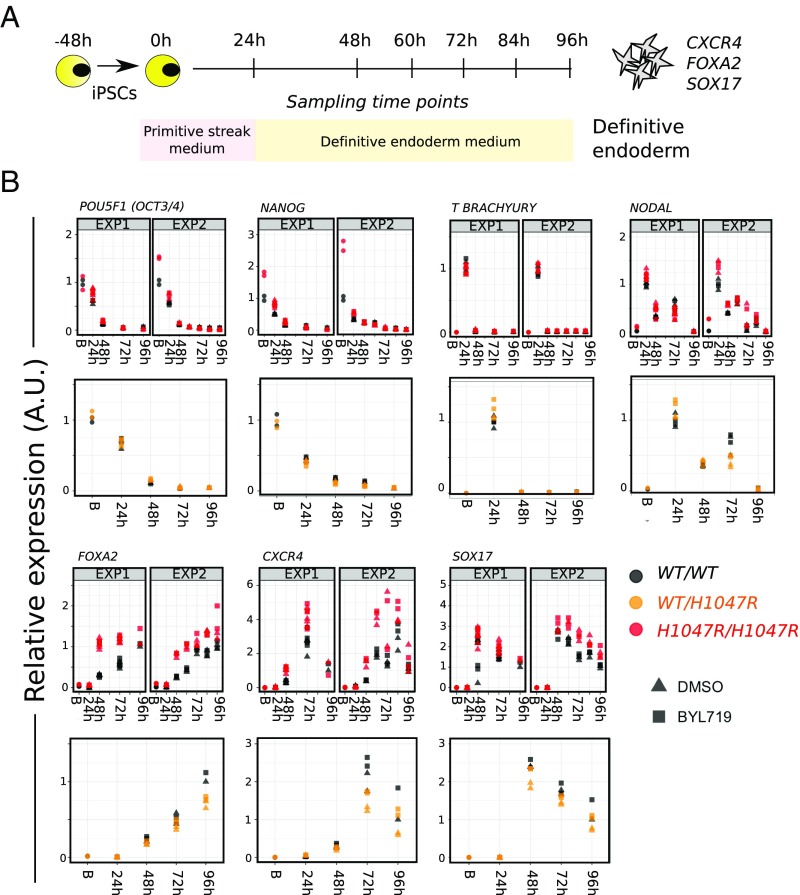
*PIK3CA*^*H1047R*^ is compatible with monolayer definitive endoderm differentiation. (*A*) Schematic of the protocol for definitive endoderm differentiation in monolayer culture. (*B*) Real-time quantitative PCR analysis of lineage marker expression during differentiation in the presence of DMSO (control) or the p110α-specific inhibitor BYL719 at 100 nM. Data from two independent experiments (EXP) with WT (*WT/WT*) vs. *PIK3CA*^*H1047R/H1047R*^ (*H1047R/H1047R*) are shown side by side (60- and 84-h time points were only assessed once). Two cultures of each of two clones per genotype were profiled. The time course data for *PIK3CA*^*WT/H1047R*^ (*WT/H1047R*) vs. WT cells are from a single experiment using two cultures of one clone per genotype. Gene expression was scaled internally to the mean value of an appropriate time point, and resulting values are arbitrary. B, baseline (0 h). See also *SI Appendix*, Fig. S7.

We also subjected WT and *PIK3CA*^*H1047R*^-harboring cell lines to monolayer-based directed differentiation using a combination of low serum, inhibition of GSK3, and high levels of Activin A (45) (Fig. 6*A*). The differentiation medium was also supplemented with DMSO (control) or BYL719 (100 nM), in anticipation that high PI3K signaling would be incompatible with 2D definitive endoderm formation, as reported previously (46, 47). Unexpectedly, both *PIK3CA*^*WT/H1047R*^ and *PIK3CA*^*H1047R/H1047R*^ iPSCs differentiated successfully to definitive endoderm under these directed conditions, as evidenced by gene expression analysis and immunostaining (Fig. 6*B* and *SI Appendix*, Fig. S7*A*). The dynamics of gene expression were closely similar across the three genotypes and were unaffected by p110α inhibition (Fig. 6*B*). Confirming that this was not a donor-specific effect, similar results were obtained with isogenic WT and mutant iPSCs derived from a PROS patient with mosaic, heterozygous expression of the rare *PIK3CA*^*E418K*^ allele (*SI Appendix*, Fig. S7*B*).

Overall, these findings suggest that PI3K activation is compatible with definitive endoderm formation in vitro, contrary to previous conclusions based on the use of nonspecific pan-PI3K inhibitors with known off-target effects (46, 47), and do not support cell-autonomous negative selection in early endoderm specification in PROS.

### Allele Dose-Dependent Effects of *PIK3CA*^*H1047R*^ in Vivo.

**Fig. 7.**
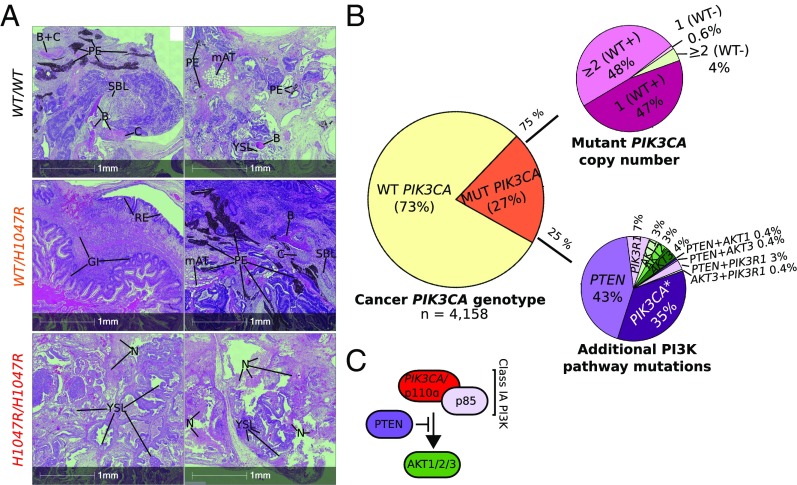
*PIK3CA*^*H1047R*^ allele dose-dependent effects in tumor xenografts and genetic evidence for graded PI3K activation in cancers. (*A*) Hematoxylin and eosin (H&E)-stained sections of WT (*WT/WT*), *PIK3CA*^*WT/H1047R*^ (*WT/H1047R*), and *PIK3CA*^*H1047R/H1047R*^ (*H1047R/H1047R*) tumor xenografts derived from injection of hPSCs into immunodeficient mice. The micrographs are from two tumors per genotype and are representative of totals of five, three, and two tumors from WT, *PIK3CA*^*WT/H1047R*^, and *PIK3CA*^*H1047R/H1047R*^ iPSCs, respectively. Yolk sac-like (YSL) and embryonal carcinoma-like (ECL) tissues, suggesting neoplastic transformation of cells within the original cultures, were more prevalent in *PIK3CA*^*H1047R/H1047R*^ tumors, which also exhibited extensive necrosis (N); rare YSL foci were seen in two other tumors derived from the same WT clone. The only well-differentiated tissue observed in *PIK3CA*^*H1047R/H1047R*^ tumors was a focus of immature bone (B) in one. WT and *PIK3CA*^*WT/H1047R*^ tumors, in contrast, comprised variable admixtures of well-differentiated and organized tissue derivatives of all three germ layers. GI, gastrointestinal tissue; mAT, mouse adipose tissue (confirmed by independent mouse vs. human immunostaining with Cyclophilin A; *SI Appendix*, Fig. S8 *A* and *B*); PE, pigmented epithelium; RE, respiratory epithelium; SBLs, sebaceous-like tissue. See also *SI Appendix*, Fig. S8 and Table S1. (*B*) The Cancer Genome Atlas (TCGA) was used to extract genomic data from *PIK3CA*-associated cancers. These were analyzed in aggregate for the presence or absence of mutant *PIK3CA* alleles, followed by stratification of *PIK3CA* mutant-positive samples based on the presence of multiple mutant alleles, including cases where the WT *PIK3CA* allele is lost (WT−). Alternatively, *PIK3CA* mutant-positive samples were screened for multiple distinct *PIK3CA* mutations (*) or for the presence of additional mutations in proximal PI3K pathway components. (*C*) Schematic of proximal class IA PI3K signaling of relevance to the analysis in *B*.

To confirm that allele dose-dependent effects of *PIK3CA*^*H1047R*^ were not artifacts of in vitro culture, we injected immunodeficient mice with WT or mutant iPSCs, and allowed them to form tumors over 5–8 wk before histopathological assessment. WT and *PIK3CA*^*WT/H1047R*^ tumors contained differentiated components of the three germ layers, including bone, cartilage, pigmented epithelium, nervous tissue, and tubular endodermal structures (Fig. 7*A* and *SI Appendix*, Table S1). All *PIK3CA*^*WT/H1047R*^ tumors exhibited better differentiated endoderm-derived tissues including respiratory (all lines) and gastrointestinal (one line) epithelium, corroborating the in vitro finding that heterozygosity for *PIK3CA*^*H1047R*^ does not impair definitive endoderm formation. In contrast, differentiated components were either completely absent or very immature in the two *PIK3CA*^*H1047R/H1047R*^ tumors (Fig. 7*A* and *SI Appendix*, Table S1), consistent with the inability of the parental cells to yield spontaneously differentiated EBs. The least mature of the *PIK3CA*^*H1047R/H1047R*^ tumors showed extensive recruitment of mouse stromal cells, forming septae separating lobules of immature human tissue (*SI Appendix*, Fig. S8*A*). Homozygous tumors also contained multiple foci positive for T BRACHYURY (immature mesoderm) and nuclear OCT3/4 (embryonal carcinoma marker in germ cell tumors) (*SI Appendix*, Fig. S8 *C* and *D*). This was further confirmed by immunohistochemistry for another embryonal carcinoma marker, CD30, which overlapped with OCT3/4-positive regions (*SI Appendix*, Fig. S8*E*). Additionally, *PIK3CA*^*H1047R/H1047R*^ tumors exhibited extensive necrosis and yolk sac-like tissue formation (Fig. 7*A* and *SI Appendix*, Fig. S8 *D* and *E* and Table S1), the latter suggested to be an in vivo characteristic of injected pluripotent stem cells with malignant potential (48). These results are in line with our in vitro studies and demonstrate that homozygosity but not heterozygosity for *PIK3CA*^*H1047R*^ promotes stemness of hPSCs.

Stem cells share many similarities with cancer cells, and phenotypes such as dedifferentiation and reactivation of developmental pathways have been linked to epithelial-to-mesenchymal transition and aggressive tumor behavior in vivo (49). *PIK3CA* mutations in human tumors are not mutually exclusive with other oncogenic alterations promoting PI3K pathway activation, suggesting that further activation is positively selected for (50). This raises the possibility that our findings may be relevant to understanding the behavior of human cancer. We thus analyzed the prevalence of multiple oncogenic “hits” within the PI3K pathway using data from The Cancer Genome Atlas (TCGA) on cancer types with >10% prevalence of *PIK3CA* mutations. In aggregate, 21% of these cancers had *PIK3CA* mutations. Nearly 40% of this subset had more than one copy of the mutation, and 25% also had a mutation in other selected PI3K pathway components (*PTEN*, *PIK3R1*, *AKT1/2/3*) or harbored a second *PIK3CA* variant (Fig. 7 *B* and *C*). This high frequency of additional mechanisms activating PI3K signaling in cancers provides circumstantial support for the notion that the strength of PI3K hyperactivation may be important for tumor progression in vivo.

## Discussion

We present a pluripotent stem cell model permitting assessment of the consequences of selective genetic p110α activation specifically in a human developmental context. By using CRISPR-mediated knockin of *PIK3CA*^*H1047R*^ into one or both endogenous *PIK3CA* alleles, we were able to examine the importance of mutant *PIK3CA* allele dosage for pathway activation and downstream cellular responses in human iPSCs. hPSCs are useful not only for study of human embryogenesis but also of the effects of pathological PI3K signaling, as seen in PROS and cancer cells (51). The model we have generated may thus be useful for understanding oncogenic actions of *PIK3CA*^*H1047R*^ in different contexts. By using expression from endogenous loci, by studying multiple clones of each genotype, and by controlling for nonspecific variation introduced during the targeting process, we have minimized analytic problems arising from overexpression of the gene of interest and from nonspecific genetic and chromosomal abnormalities.

*PIK3CA*^*H1047R*^ increased PI3K signaling “tone” both in growth factor-replete and growth factor-depleted medium. Most strikingly, we report distinct allele dose-dependent effects of mutant *PIK3CA* on stemness and pluripotency in vitro and in vivo, with a corresponding major alteration of the transcriptome triggered at a threshold between heterozygous and homozygous p110α activation. At odds with our finding in human stem cells, heterozygous expression of *PIK3CA*^*H1047R*^ in a human MCF10A breast epithelial cell line has previously been shown to cause widespread transcriptional changes, illustrating the notion that small changes in a nonlinear system can have extensive consequences (52, 53). However, the mutant cells in these studies also had amplification of chromosome 5p13-15 (53), a region harboring the gene encoding the catalytic subunit of telomerase. This could have contributed to the observed discrepancy to our study. Alternatively, thresholds at which p110α signaling triggers its transcriptional effects may differ among cell types. Exemplifying this, either overexpression or endogenous expression of *PIK3CA*^*H1047R*^ induces multipotency in mammary tumors (8, 16), with the tumor cell of origin dictating phenotypic severity.

The generation of human developmental cell models of *PIK3CA*^*H1047R*^ is also important given the well-documented differences between the pathways regulating mouse and human stem cell pluripotency and differentiation (54). Although we describe a stem cell-based study focusing on endogenous expression of the commonest pathogenic *PIK3CA* allele, several other studies have adopted different strategies to activate other components of the PI3K/AKT signaling cascade in this cell type (40, 55–58). Self-sustained stemness is a common motif in the phenotypes reported, and some studies, like ours, argue for discernible PI3K dose dependency. For example, mouse pluripotent stem cells with homozygous knockout of the isoform-agnostic type IA PI3K negative regulator *Pten* exhibit impaired differentiation in vitro and in vivo, but this is not seen in heterozygous knockout cells (57). How strong PI3K activation sustains stemness remains to be determined; however, our data suggest that induction of TGFβ signaling via NODAL is likely to be important. Supporting this, several transcriptional changes observed in *PIK3CA*^*H1047R/H1047R*^ cells were reciprocal to those in hPSCs exposed to pharmacological inhibition of TGFβ signaling (59). It is also possible that the direct link between PI3K activation and *NODAL* expression underlies the previously reported association between PI3K/AKT activation and expression of *NANOG* (56, 60), a key pluripotency gene controlled by SMAD2/3 (61).

In contrast to the complex genetics of cancer, activating *PIK3CA* mutations arise heterozygously and in isolation in the severe overgrowth disorders known as PROS. An excess risk of adult cancer has not been reported in these mosaic disorders, in line with accumulating evidence that heterozygosity for *PIK3CA*^*H1047R*^ alone is not sufficient to cause cellular transformation (5). PROS also illustrates the importance of controlled p110α signaling in early human development. Overgrowth in PROS commonly affects mesodermal and neuroectodermal lineages but rarely endoderm-derived tissues, prompting speculation that a sustained increase in PI3K activation impairs endoderm development (5). It has also been reported that class IA PI3K signaling is incompatible with directed definitive endoderm formation from hPSCs, although this assertion is largely based on use of nonspecific pan-PI3K inhibitors (46, 47). In our study, we found no evidence that genetic PI3K activation impairs guided definitive endoderm formation in culture. Moreover, *PIK3CA*^*WT/H1047R*^ pluripotent stem cells gave rise to teratomas featuring well-differentiated endodermal components, arguing against a cell-autonomous defect in endoderm specification as an explanation for overall lack of endodermal overgrowth in PROS. The relatively mild biochemical and transcriptional consequences of heterozygous *PIK3CA* activation in stem cells, and their grossly normal early differentiation in several different experimental contexts, suggest that any negative selection in certain lineages may be exerted only at later stages of differentiation. In contrast, homozygosity for *PIK3CA*^*H1047R*^ in early development will likely be selected against due to impaired differentiation and embryonic lethality.

For all of the modesty of the cellular effects and lack of increased adult cancer risk in PROS, we emphasize that heterozygosity for *PIK3CA*^*H1047R*^ is unequivocally causal in PROS, reflecting the cumulative effects of sustained low-grade growth promotion over an individual’s lifetime. The relatively small signaling perturbation conferred by *PIK3CA*^*H1047R*^ heterozygosity, and the lack of cooperating lesions, makes treatment with a low-dose p110α inhibitor a particularly promising option in this setting. Consistent with this, low-dose BYL719 was shown recently to produce highly clinically significant regression of overgrowth in adults and children with PROS, without the side effects associated with PI3K inhibition in cancer trials (62).

Our report of marked allele dose-dependent effects of *PIK3CA*^*H1047R*^ may also have implications for understanding of PI3K-associated cancers. Many human cancers feature oncogenic alterations in *PIK3CA*, and not only do these often occur with mutations in other pathway components, but our data demonstrate the frequent presence of more than one mutant *PIK3CA* copy, suggesting that cancer cells benefit from additional PI3K pathway activation. Future studies of the role of the PI3K pathway in cancer progression should incorporate consideration of PI3K signaling “dose” and the possibility of clear thresholds for biological consequences. Such considerations echo recent reports that an increased dosage of mutant *KRAS* influences clinical outcome and therapeutic targeting (63, 64). Supporting this notion, Bielski et al. (65) also found that oncogene allelic imbalances in human cancers were selected for through modest dosage increases of gain-of-function variants, with consequences for sensitivity to targeted therapy. Their study provides systematic evidence from human cancers against the commonly held view that gain-of-function mutations in cellular oncogenes are typically heterozygous, where a dominant mechanism of action is thought sufficient to promote oncogenesis. Our genomic analyses focusing on *PIK3CA*-associated cancers and oncogenic “hits” within the PI3K pathway, combined with direct cellular evidence of allele dose-dependent effects of *PIK3CA*^*H1047R*^, adds further support to a revised oncogene model that takes into account the functional implications of allelic imbalances. Based on these observations, it will be interesting to determine whether cancers with stronger activation of PI3K exhibit more aggressive features such as a higher degree of dedifferentiation and metastatic potential. Conversely, therapeutic sensitivity may also be higher in tumors with increased PI3K signaling dose. Of note, a recent clinical study evaluating the efficacy of AKT inhibition in patients with the *AKT1*^*E17K*^ mutation found frequent homozygosity for this variant, and this was associated with a statistically and clinically significant improvement in therapeutic response (66). As the authors note, this may suggest that future patient stratification for targeted cancer therapy should take into account the tumor’s genomic configuration (66), including differences in oncogene dosage and coincident oncogenic “hits” within the same pathway.

In summary, our study demonstrates that the cellular consequences of the most common oncogenic *PIK3CA* mutation are allele dose dependent. The observed near binary differences between *PIK3CA*^*H1047R*^ heterozygosity and homozygosity suggest that cells may have a PI3K signaling threshold that determines the pathological consequences of this variant in development and cancer. Prospective clinical studies are needed to determine whether differences in the allele dosage of activating *PIK3CA* mutations influence therapeutic outcomes in cancer.

## Methods

Additional information, including reagent catalog numbers and nucleic acid sequences, are provided in *SI Appendix*.

### Experimental Models.

CRISPR/Cas9 targeting was performed on the male WTC11 iPSC line line, a kind gift from Bruce Conklin (Gladstone Institutes and University of California, San Francisco). The derivation of this line has been described (67), and publicly available RNA, whole-exome, and whole-genome sequencing data are available via the Conklin laboratory’s website (https://labs.gladstone.org/conklin/wtc-information.html) or via the Coriell Institute (GM25256). In the current work, the parental line was used for gene editing at passage numbers P37 and P38. The derived iPSCs were used for experiments between P45 and P60.

The PROS patient-derived iPSC lines M98-WT and M98-E418K were obtained from a female, 18-y-old PROS patient by episomal reprogramming of a dermal fibroblast culture with 32% mosaicism for *PIK3CA*^*E418K*^. All clones used for experimental studies were confirmed transgene-free and expressed high levels of PSC-specific markers, comparable to those of a reference hPSC line. Karyotyping on a single line from each genotype confirmed lack of microscopic genetic rearrangements. The original patient-derived dermal fibroblasts were obtained with full informed consent in accord with the Declaration of Helsinki. The study was approved by The Cambridge South Ethics Committee (study reference no. 12/EE/0405).

### CRISPR/Cas9 Targeting of Human iPSCs.

The WTC11 iPSC line was targeted with plasmid-delivered WT Cas9 (pX459; Addgene; 48139) and gBlock-encoded FE-modified single guide RNAs (sgRNAs) (68). Targeting was performed by nucleofection of 5 μg of pX459 plasmid (Cas9 WT), 3 μg of sgRNA-encoding gBlock, and either 200-pmol targeting template (for homozygous targeting) or a combination of 100-pmol targeting and “mock” templates (for heterozygous targeting). The nucleofected cells were seeded into Geltrex-coated 96-well plates and processed for sib-selection when ready for passaging. Sib-selection was performed as described previously (69), using 25–100 cells per well in each subcloning round. WT iPSC lines obtained in the process of subcloning were banked as genetically matched controls. Genotyping, including off-target assessment, by Sanger sequencing and restriction fragment length polymorphism assays are described in *SI Appendix*.

### Differentiation Assays.

#### EBs.

EBs were established either by spontaneous self-aggregation of hPSCs or by forced aggregation into AggreWell plates. For self-aggregation, 50–70% confluent hPSCs were dissociated into aggregates with ReLeSR, and the entire cell suspension from a six-well transferred to one 60-mm Nunclon Sphera ultra-low attachment dish in Essential 6 (E6) medium supplemented with 0.4% (wt/vol) polyvinyl alcohol (PVA) and RevitaCell (E6/PVA+R). EBs formed within 24 h, after which the medium was exchanged with E6 (without PVA and RevitaCell). The medium was exchanged again on day 3 of EB formation. For adherent outgrowths, the EBs were transferred to Geltrex-coated six-well plates on day 4, either in regular E6 or in E6 supplemented with 10% (vol/vol) FBS, 100 nM BYL719, or 0.01% (vol/vol) DMSO. The EBs from a single Nunclon Sphera dish were used to seed four wells of a 6-well plate or eight wells of a 12-well plate. EB outgrowths were collected for RNA extraction on day 10 of EB formation. In one experiment, suspension EBs were also collected on day 4 and day 13.

EB set-up in AggreWell plates followed the manufacturer’s instructions, with E6/PVA+R as medium for cell seeding. A total of 2.4 × 10^5^ cells was seeded in each well, for a final density of 200 cells per microwell. EBs formed within 24 h, and the contents of four to five individual wells were transferred to a single Nunclon Sphera ultra-low attachment dish for culturing in either mesoderm (10 ng/mL BMP4, 5 ng/mL Activin A, 5 ng/mL FGF2) or endoderm (0.25 ng/mL BMP4, 100 ng/mL Activin A, 2.5 ng/mL FGF2) induction medium. After 3 d of induction, the EBs were transferred to Geltrex-coated six-well plates for adherent growth and maintained in E6 until day 10. Cells were collected for RNA extraction on day 0 (iPSC stage), day 4, day 7, and day 10 of EB formation. For immunocytochemistry, day 4 EBs were also seeded for adherent growth in Geltrex-coated four-well or 35-mm Ibidi imaging dishes and processed for staining on day 10.

#### Definitive endoderm differentiation.

Definitive endoderm differentiation of iPSCs was carried out according to a modified version of the protocol described in ref. 45. Further details are provided in *SI Appendix*.

### Tumor Xenograft Assays.

Tumor xenografts were generated from a total of 10 iPSC cultures (6 WT, 3 *PIK3CA*^*WT/H1047R*^, 2 *PIK3CA*^*H1047R/H1047R*^) by s.c. injection into immunodeficient, male NSG mice (005557; The Jackson Laboratory) at 12 wk of age. Individual animals were culled when tumors reached ∼1.4 cm^3^ in size, or if they became ill suddenly. All animal procedures were performed with approval from the local Animal Welfare Ethical Review Body and in accordance with Home Office regulations [The Animal (Scientific Procedures) Act 1986].

Each tumor was processed for formalin fixation, paraffin embedding, microtome sectioning, and hematoxylin and eosin (H&E) staining as described in ref. 70. The slides were analyzed blindly by a human pathologist and processed for automated bright-field imaging on an AxioScan Z1 (Zeiss) slide scanner.

### RNA Sequencing.

A total of 1 µg of RNA per sample was used to synthesize 50-bp-long single-end mRNA libraries with an Illumina TruSeq Stranded mRNA Library Prep Kit. The integrity and quantity of the libraries were determined on the Bioanalyzer using the DNA 12000 Kit (Agilent). The barcoded libraries were pooled and sequenced on an Illumina HiSeq 4000, with an average depth of 20 million reads per sample. The raw reads were mapped to the human genome build GRCh38, and gene level counts were determined using Spliced Transcripts Alignment to a Reference, version 2.5 (71). Subsequent data processing followed the method outlined in ref. 72.

### TCGA Data Analysis.

The cancer genome analyses presented in this work are based upon data generated by the TCGA Research Network: https://cancergenome.nih.gov/. Somatic mutation tables (minor allele frequencies) from whole-exome sequencing data across 11 cancer types (BLCA, BRCA, CESC, CRC, ESCA, GMB, HNSC, LUSC, STAD, UCEC, and UCS) were downloaded from the TCGA portal through the Genomic Data Commons Data Transfer Tool. Mutation calls generated by Varscan2 (73) were used. To limit false positives, for those variants with a VAF (t_alt_count/t_depth) < 0.05, we retained those that were also identified by the MuTect2 algorithm (74). Functional annotation of genomic variants was performed with ANNOVAR (75). Purity, ploidy, and copy number profiles of tumor cells were obtained with ASCAT (76) run using default parameters on SNP6.0 data. For additional details, see *SI Appendix*.

## Supplementary Material

Supplementary File
