# Supplementary material for: Oncogenic PIK3CA promotes cellular stemness in an allele dose-dependent manner
Source: Proc Natl Acad Sci U S A. 2019 Apr 4;116(17):8380–9. doi: 10.1073/pnas.1821093116 (PMC6486754; doi:10.1073/pnas.1821093116)
Supplement: Supplementary File [file pnas.1821093116.sapp.pdf]

PNAS
www.pnas.org

Supplementary Information for

Oncogenic *PIK3CA* promotes cellular stemness in an allele dose-dependent manner

Ralitsa R. Madsen, Rachel G. Knox, Wayne Pearce, Saioa Lopez, Betania Mahler-Araujo, Nicholas McGranahan, Bart Vanhaesebroeck, Robert K. Semple

Robert K. Semple
Email: rsemple@ed.ac.uk

This PDF file includes:

- Supplementary Methods
- Figs. S1 to S8
- Tables S1 to S9
- SI reference citations

Supplementary Methods

Unless stated otherwise, catalogue numbers and vendor details of reagents used in this work can be found in Table S9.

Cell culture

Cells were grown at 37 °C and 5 % CO_2 in Essential 8 Flex (E8/F) medium on Geltrex-coated plates, in the absence of antibiotics. For maintenance, 70-90 % confluent cells were passaged as aggregates with ReLeSR, in the presence of the ROCK inhibitor RevitaCell (E8/F+R) during the first 24 h to promote survival. For experiments that required precise control of cell numbers, iPSC colonies were dissociated into single cells with StemPro Accutase, prior to manual cell counting.

All cell lines were tested negative for mycoplasma and genotyped routinely to rule out cross-contamination during prolonged culture. STR profiling was not performed.

Additional details on the cell lines used in this work are provided in Table S2.

Preparation for xenograft assays

For tumor xenograft assays, cells were cultured according to standard procedures in Geltrex-coated T75 flasks, ensuring 95-100 % confluence on the day of injection. The cells were processed for aggregate dissociation with ReLeSR, collected in E8/F+R and centrifuged at 200g for 3 min. Each cell pellet was resuspended in 130 µl ice-cold E8/F+R, followed by mixing with 70 µl ice-cold human ESC-qualified Matrigel. From this suspension, 200 µl were used for injections within 30 min of preparation (kept on ice throughout), using pre-chilled syringes (20.5 gauge needles).

Karyotyping

For karyotyping, subconfluent iPSCs from two 6-wells were treated with 0.1 µg/ml KaryoMAX Colcemid solution for 90 min, dissociated into single cells, and the final cell pellet resuspended in hypotonic KCl solution (0.055 M) for 5 min at 37 °C. Next, the suspension was centrifuged at 200g for 5 min, and the pellet resuspended in freshly prepared cold fixative (3:1 methanol:glacial acetic acid). The fixed cell suspension was used for slide preparation and banding analysis by the East Anglian Medical Genetics Service.

Genotyping

For routine, confirmatory genotyping and for rapid post-gene editing mutation screening, genomic DNA (gDNA) from cells was extracted using QuickExtract solution according to the manufacturer's instructions. For high-quality sequencing, including screening for off-target and *TP53* mutations, gDNA was extracted with Qiagen's QIAamp DNA Micro Kit according to the manufacturer's instructions. The targeted DNA region was amplified by PCR using pre-made GoTaq Green mastermix according to the manufacturer's protocol, and the amplicon sequenced in both directions using the BigDye Terminator v3.1 Cycle Sequencing Kit following the manufacturer's protocol.

For restriction fragment length polymorphism (RFLP)-based genotyping, the GoTaq Green PCR reaction was performed with a FAM-labelled version of the reverse primer. From the 25 µl PCR reaction, 10 µl were taken forward to a 30 µl digestion reaction with BmgBI, performed in line with the manufacturer's protocol. Next, 2 µl of each digestion reaction were mixed with 13 µl GeneScan 600 LIZ size standard stock, followed by capillary electrophoresis and fluorescent detection on an ABI 3730 sequencer (Thermo Fisher Scientific). The standard stock was prepared by mixing 1 ml HiDi formamide with 45 µl LIZ standards. Following electrophoresis, the results were extracted using the GeneMapper 5.0 software. The percentage of *PIK3CA*^{H1047R}-positive cells was determined from individual peak areas with the following formula:

$$E = \text{(area of target peak)} / [\text{(area of target peak)} + \text{(area of unmodified peak)}]$$

Where target peak corresponds to the signal from the BmgBI digestion product.

All genotyping primer sequences are provided in Table S4.

Off-target screening

Off-target sites were analyzed with the Zhang Lab's online sgRNA tool (October 2015). With the development of more advanced algorithms, the off-target list was re-evaluated in September 2017 using the Cas-OFFinder algorithm (1), selecting NGG as PAM site and allowing for up to five mismatches and two bulges against the human genome build hg38. The resulting list was filtered for targets with up to three mismatches, including up to two mismatches with two bulges. The top 15 off-target sites (both coding and non-coding) predicted by Cas-OFFinder and the three coding off-target sites (among all top 50 off-targets) obtained with the Zhang Lab's tool were selected for confirmatory genotyping by Sanger sequencing in three WT clones, three $PIK3CA^{WT/H1047R}$ clones and six $PIK3CA^{H1047R/H1047R}$ clones. Additionally, a tiled primer matrix was designed, spanning 851 bp and 1126 bp upstream and downstream, respectively, of *PIK3CA* codon 1047. This was used to test for larger DNA rearrangements that could arise from CRISPR targeting (2). Arguing against such rearrangements in our cells, different primer combinations gave rise to an amplification product of the correct size in the profiled WT (n = 1), $PIK3CA^{WT/H1047R}$ (n = 3) and $PIK3CA^{H1047R/H1047R}$ (n = 10) clones. In addition, all PCR products from the WT clone and two $PIK3CA^{H1047R/H1047R}$ clones were Sanger sequenced, confirming lack of unintended indels.

As an additional check that the observed homozygosity for $PIK3CA^{H1047R}$ was not a false positive caused by loss of heterozygosity in the targeted cells, gDNA from WT and $PIK3CA^{H1047R/H1047R}$ iPSCs were mixed 1:1 and sequenced across *PIK3CA* codon 1047. This resulted in equally-sized chromatogram peaks for the WT and $PIK3CA^{H1047R}$ allele, consistent with homozygosity in the mutant cell lines. This check was performed with 7 $PIK3CA^{H1047R/H1047R}$ clones, mixed with either one of two independent WT clones.

The relevant primer sequences are provided in Table S4.

Definitive endoderm differentiation

Definitive endoderm differentiation of iPSCs was carried out according to a modified version of the protocol described in (3). Isogenic lines were seeded in Geltrex-coated 12-well plates at densities between 5,000-7,500 cells/cm^2 (WT, $PIK3CA^{WT/H1047R}$, $PIK3CA^{H1047R/H1047R}$) or 8,500 cells/well^2 (WT, $PIK3CA^{WT/E418K}$), seeding a minimum of two cultures per clone. Two days post-seeding, the cells were induced to differentiate, in the presence of BYL719 (100 nM) or the corresponding dilution of DMSO. Samples were collected for RNA extraction at baseline (immediately before induction) and on each one of the following days of the differentiation protocol. In one experiment, cells were also fixed for immunofluorescence at the end of differentiation.

RNA processing and RT-qPCR

Cell lysates were processed for RNA extraction either with the DirectZol Kit or the RNeasy Mini Kit as per the manufacturer's instructions. Live or snap-frozen cells in 12- or 6-well plates were lysed in 500-750μl QIAzol or RLT buffer (supplemented with 10 % (v/v) β-mercaptoethanol). The final RNA was quantified on a NanoDrop ND-1000 and quality assessed by agarose gel electrophoresis. Samples showing signs of RNA degradation were excluded from further analysis. The remaining samples were diluted to 20-100 ng/μl and 200-1000 ng used for reverse transcription (RT) with the High-Capacity cDNA Reverse Transcription Kit according to the manufacturer's instructions. For RNAseq, total RNA was extracted as described above, followed by quantification and quality assessment on an Agilent Bioanalyzer using the RNA 6000 Nano Kit according to the manufacturer's instructions, confirming that all samples had a RIN score of 10.

For downstream quantitative PCR (qPCR), the cDNA samples were diluted to a final concentration of 1-2.5 ng/μl. Duplicate qPCR reactions were set up in 384-well plates using 2-5 ng/μl cDNA per reaction and SYBR Green chemistry. The samples and the appropriate lineage-specific cDNA standards were run on a Quant Studio 7 Flex Real-Time PCR System (Thermo Fisher Scientific) using the default settings for SYBR reagents, with melting curve acquisition and standard run properties. The thermal conditions were as follows: 2 min at 50 °C; 10 min at 95 °C; 40 cycles at 95 °C

for 15 s and 60 °C for 1 min; all with a ramping rate of 1.6 °C/s. Following data acquisition, a mean Ct value was calculated for each sample by taking the average of the technical replicates. Samples were re-run when the technical replicates yielded discordant Ct values (> 0.6 Ct difference), except in cases where this could be attributed to low expression of the targeted gene (e.g. differentiation genes in iPSC samples and stemness genes in differentiated samples). The relative expression value of each gene was determined from the corresponding cDNA standard curve (4). Gene expression values were set to 0 if the Ct values were below the limit-of-detection (LOD) of the standard curve, or within 5 Ct values from the corresponding -RT control(s) when using non-intron-spanning primers. The expression values of individual genes were normalized to the corresponding *TBP* (TATA-binding protein) values, following confirmation that *TBP* expression did not change across conditions. Within each experimental replicate, the final gene expression values were scaled internally to the mean of an appropriate condition to make results comparable across individual experiments.

SYBR Green qPCR primer sequences are included in Table S5.

TaqMan hPSC Scorecards

TaqMan hPSC Scorecards (384-well) were used according to the manufacturer's protocol and run on the Quant Studio 7 Flex system. The Ct values were linearized by applying antilog2, based on the assumption of 100 % amplification efficiency. Genes were excluded from analysis if their expression was low (Ct ≥ 30) across all samples. The seven control gene assays on each plate were used to calculate a geometric mean, which was used for normalization of the remaining gene expression values.

RNAseq pathway analysis

Ingenuity® Pathway Analysis (build version 448560M, content version 36601845) was conducted on the 1,914 differentially expressed genes in $PIK3CA^{H1047R/H1047R}$ iPSCs, using the Ingenuity Knowledge Base (Genes Only) as reference set and including both direct and indirect relationships. Relationships were only considered if experimentally observed. The results from the Upstream Regulator Analysis (5) were exported and processed for visualization in RStudio. Independently, the same set of genes were used for pathway analysis with the CytoScape plug-in ClueGO (v2.3.4), focusing on pathways within the KEGG ontology (Build 01.03.2017) and applying the following parameters: merge redundant groups with 50 % overlap; evidence codes used: all; statistical test: enrichment/depletion (two-sided hypergeometric test) with Benjamini-Hochberg correction; minimum number of genes per term = 10; minimum percentage enrichment = 4; Kappa Score Threshold = 0.4. The created network represents the pathway terms as nodes, linked based on term-term similarity as determined by the Kappa Score. The size of the nodes reflects the enrichment significance of the terms, and only terms with FDR ≤ 0.05 are shown.

Mutation copy number analysis

Mutation multiplicity, or mutation-copy-number, describing the number of mutant copies of a mutation, is a function of the purity of the sample, the ploidy of the tumor cells and the relative frequency of the mutation (i.e. the VAF). The mutation multiplicity was calculated as previously described (6), using the following equation:

Mutation copy number = (VAF/p)*((p*CNt)+2*(1-p))

Where CNt is the local copy number at the mutated base and p is the estimated purity of the tumor sample. Mutations exhibiting a mutation multiplicity ≥ 1.5 were classified as 'gained'. If the mutation multiplicity was equal the local copy number and the genomic segment harboring the mutation was subject to loss of heterozygosity (LOH), the wild-type allele was inferred to be absent.

Immunohistochemistry (IHC)

Manual (fluorescence)

Formalin-fixed and paraffin-embedded (FFPE) teratoma sections were processed for dewaxing and rehydration, followed by high-pH antigen retrieval in DAKO Antigen Retrieval Solution (pH 9) in a water bath at 97 °C for 20 min. For Cyclophilin A-based detection of mouse host tissue (7), DAKO's Antigen Retrieval Solution (pH = 6) was used instead. Next, the slides in antigen retrieval solution were cooled down for 15 min at -20 °C, followed by a wash in Tris-buffered saline (TBS; 20 mM Trizma base, 150 mM NaCl, pH = 7.6) supplemented with 0.1 % Tween-20 (TBS/T), and two washes in DAKO wash buffer (5 min per wash). Slides were blocked in 2.5 % ready-to-use (R.T.U.) horse serum (HS) for 1 h at room temperature, using 300 µl blocking solution per slide. Primary antibody dilutions, including the corresponding isotype controls, were prepared in 2.5 % R.T.U. HS, and 200 µl were applied to each slide for overnight incubations at 4 °C. For co-staining, primary antibodies were applied simultaneously. The following day, the slides were placed at room temperature for 2 h, then washed five times in DAKO wash buffer, before room temperature incubation with the appropriate combination of secondary antibodies diluted 1:1,000 in 2.5 % R.T.U. HS. For dual staining requiring detection with goat anti-mouse and donkey anti-goat secondary antibodies, each slide was firsts incubated in donkey anti-goat antibody for 2 h, followed by five washes in DAKO wash buffer, and another 2-h incubation in goat anti-mouse antibody, thus avoiding cross-reactivity between the two secondary antibodies. The second incubation was omitted when working with a single secondary antibody. After the final incubation, the slides were washed another five times and processed for overnight mounting in Fluoroshield DAPI, carried out at room temperature. The slides were processed for automated image acquisition on the AxioScan Z1 slide scanner, and the HALO software (Indica Labs) was used for contrast/brightness adjustments and visualization. The same adjustments were applied to all images obtained in the same staining round. The specificity of each signal was determined based on its subcellular localization and a comparison to the corresponding IgG control staining. Automated cell counting of T BRACHYURY-positive cell was conducted in HALO, following nuclear segmentation based on the DAPI signal. The analysis was performed on entire tissue slides. Primary antibody details are provided in Table S6; secondary antibodies are listed in Table S9.

Automated (HRP)

FFPE slides were processed for horseradish peroxidase (HRP)-based immunohistochemistry on a BOND-III automated stainer (Leica). Details on antigen-retrieval method and antibody dilutions are provided in Table S7.

Immunocytochemistry (ICC)

Adherent cells were fixed for 15 min with 4 % (v/v) formaldehyde, followed by permeabilization in PBS supplemented with 0.1 % (v/v) TritonX-100 (PBS/T) for 15 min and 60-90 min blocking in PBS/T with 5 % (v/v) horse serum (HS; PAN-Biotech). Primary antibodies were diluted in PBS with 1 % (v/v) HS and used in overnight incubations at 4 °C. For co-staining, multiple primary antibodies were applied simultaneously. The following day, the samples were equilibrated to room temperature, washed three times in PBS and incubated with the appropriate fluorophore-conjugated secondary antibodies diluted in PBS with 1 % HS, for 2 h away from light. Co-staining with secondary antibodies with the potential to cross-react was performed as outlined in the previous section, with PBS used as wash buffer and PBS with 1 % HS as dilution buffer. Anti-rabbit and anti-mouse secondary antibodies were diluted 1:1,000, and the anti-goat antibody 1:2,000. Next, cells were incubated in CytoPainter Phalloidin-iFlour 555 (1:1,000) for 45 min for F-Actin visualization, followed by nuclear counterstaining with Hoechst, DAPI (Nuclear Select FX Labeling Kit) or NucGreen Dead as indicated in the results. The nuclear stain was removed by two or three washes in PBS, each lasting 5 min. Stained cells were either imaged directly in PBS or processed for mounting (Fluoroshield +/- DAPI or Prolong Gold Antifade). Images were captured either on an EVOS FL microscope using the 10X and 20X objectives, or on a Leica TCS SP8 confocal laser microscope using an HC PL APO 20X/0.75 dry objective (no. 15506517) or an HC PL APO 20X/0.75 oil-immersion objective (no. 15506343). The LAS X Software from Leica was used to acquire 8-bit confocal images at a resolution of 512 x 512 or 1024 x 1024 pixels, with line averaging set to 8 or 16. For each experiment, the same laser settings were used to image all samples, with the settings optimized to avoid saturation and photobleaching. Images were adjusted for brightness and contrast in FIJI ImageJ, applying the same changes to the entire dataset.

All primary antibodies used for IHC and ICC were first tested individually to confirm specificity and lack of interference in subsequent co-staining experiments. Primary antibody details are provided in Table S6; secondary antibodies are listed in Table S9.

Western blotting

For protein extraction, adherent cells were lysed in 100-150 µl (per 6-well) ice-cold protein lysis buffer containing: 50 mM HEPES, 150 mM NaCl, 1.5 mM MgCl2, 10 % (v/v) glycerol, 1 % (v/v) TritonX-100, 1 mM EGTA, 100 mM NaF, 10 mM $Na_4P_2O_7$, 2 mM Na_3VO_4 (added fresh), 1X EDTA-free protease inhibitor tablet (added fresh), 1X PhosStop tablet (added fresh). Protein concentrations were measured using BioRad's DC protein assay, and all concentrations were adjusted to 1 mg/ml with lysis buffer and LDS sample buffer supplemented with 1X Reducing Agent or 2.5 % (v/v) β-mercaptoethanol. Following denaturation at 70 °C for 10 min, 10 µg of each sample were resolved on NuPAGE 4-12 % gradient gels, using MOPS running buffer supplemented with NuPAGE antioxidant. Next, proteins were transferred to nitrocellulose membranes on an iBlot device (Thermo Fisher Scientific) using program 3 (P3; 20 V) for 7-10 min depending on protein size. Following transfer, the gels were stained with Coomassie (EZ Blue) to assess transfer efficiency and loading accuracy. The nitrocellulose membranes were blocked for 60-90 min in 3 % (v/v) BSA in TBS/T. This was followed by overnight incubation (4 °C) in primary antibody diluted in blocking buffer, up to eight washes in TBS/T and room temperature incubation in horseradish peroxidase (HRP)-conjugated secondary antibodies (diluted 1:10,000) for 1-2 h. The final blots were detected by enhanced chemiluminescence (ECL), using the Immobilon Western Chemiluminescent HRP Substrate and a chemiluminescence imaging analyzer (LAS4000 mini, Fujifilm). Images were processed for cropping and contrast adjustments using FIJI ImageJ (https://imagej.nih.gov/ij/). Densitometry analyses of selected blots were carried out in ImageStudioLite (LI-COR) Version 5.2.5, using the "median" method for background correction.

Details on primary antibodies used for Western blotting are provided in Table S8; secondary antibodies are listed in Table S9.

Annexin V apoptosis assay

Apoptosis was assessed with an Annexin V-Cy5 Apoptosis Staining Kit according to a modified version of the manufacturer's protocol. To limit apoptosis due to enzymatic dissociation, the cells were detached with ReLeSR for 15 min prior to collection. The conditioned growth medium and all PBS washes were collected along with the dissociated cells. All samples were centrifuged at 4 °C and 200g for 3 min and the supernatant discarded. Each cell pellet was resuspended in 3 ml ice-cold PBS, followed by repeated centrifugation. The final pellet was resuspended in 250 µl ice-cold 1X Binding Buffer and transferred directly to a FACS tube with a cell strainer lid, allowing the cell suspension to pass through by capillary action. Next, 2.5 µl Annexin Stain were added to each sample, followed by a 20-min incubation on ice prior to fluorescence acquisition. A spare cell suspension was left unstained and used as negative control. A suspension of dead cells was used as positive control. Fluorescence measurements were acquired on a BD Accuri C6 Plus flow cytometer (BD Biosciences) at 675/25 nm with medium flow (35 µl/min) and a core size of 16 µm. At least 50,000 events were acquired per sample. The BD Accuri software was used for gating, ensuring that cell doublets were excluded and that dead, live apoptotic and healthy live cells could be separated based on cell size and Annexin V staining.

Study design and replication

A power analysis was carried out in advance of the RNA sequencing analysis, using the method described in (8). According to this analysis, three biological replicates would yield a power of 50 % to detect differentially expressed genes between two genotypes, with a minimum fold-change difference of 1.8 and assuming that 70 % of all genes remain unaltered. This was deemed sufficient for the purpose of this experiment.

"Biological replicates" refer to independent iPSC clones. Technical replicates refer to multiple cultures of the same iPSC clone, and experimental replicates refer to repeats of the same experiment on different occasions. Additional experimental replicates were included with assays characterized by high

internal variability such as spontaneous EB differentiation. The exact number of each replicate type is stated in the relevant figure legends. Randomization was not required unless in experiments with multiple treatment regiments (e.g. GF stimulations) across different genotypes; in these cases, experimental bias was minimized by treatment and genotype randomization across independent experimental replicates.

DATA PROCESSING

Data processing and visualization were performed in RStudio (https://www.rstudio.com/). All raw data and bespoke RNotebooks containing guided scripts used to analyze larger datasets are available *via* the Open Science Framework (OSF) (doi: 10.17605/OSF.IO/DVJNT). All uncropped Western blots are also provided *via* OSF; this includes both blots that are displayed in the paper as well as additional replicates. The RNAseq data discussed in this publication have been deposited in NCBI's Gene Expression Omnibus (9) and are accessible through GEO (GSE126562).

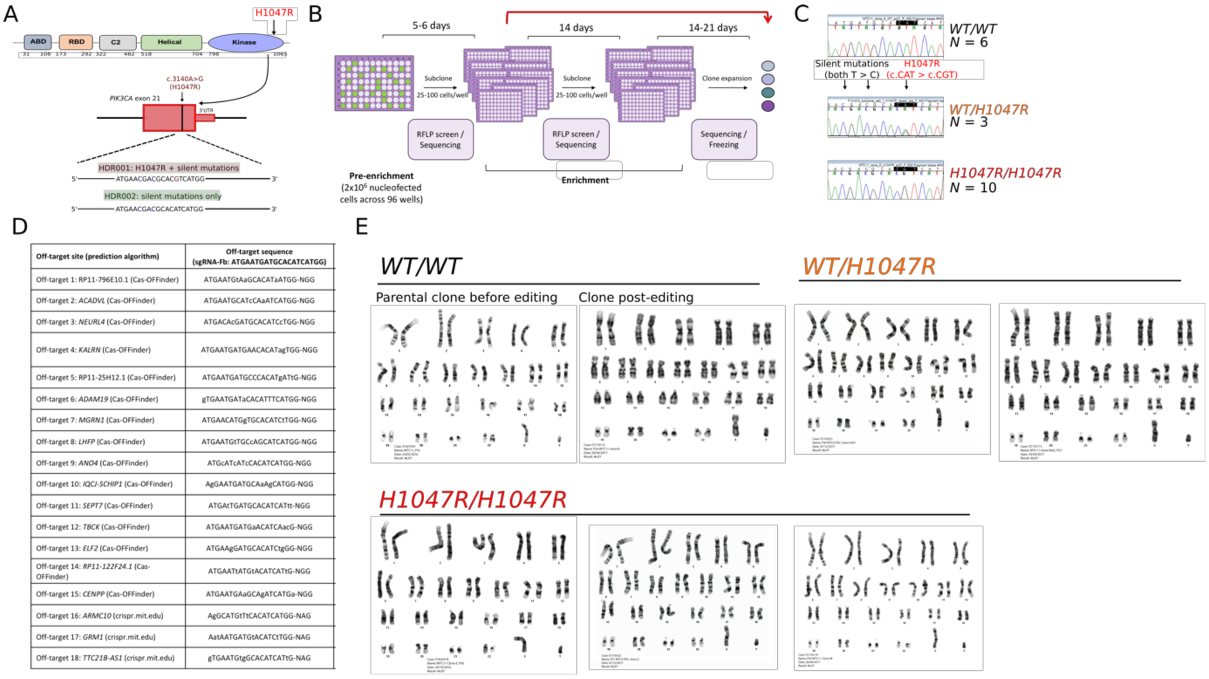

Off-target site (prediction algorithm)	Off-target sequence (sgRNA-Fb: ATGAATGATGCACATCATGG)
Off-target 1: RP11-796E10.1 (Cas-OFFinder)	ATGAATGtAaGCACATaATGG-NGG
Off-target 2: *ACADVL* (Cas-OFFinder)	ATGAATGCATcCAaATCATGG-NGG
Off-target 3: *NEURL4* (Cas-OFFinder)	ATGACAcGATGCACATCcTGG-NGG
Off-target 4: *KALRN* (Cas-OFFinder)	ATGAATGATGAACACATagTGG-NGG
Off-target 5: RP11-25H12.1 (Cas-OFFinder)	ATGAATGATGCCCACATgATtG-NGG
Off-target 6: *ADAM19* (Cas-OFFinder)	gTGAATGATaCACATTTCATGG-NGG
Off-target 7: *MGRN1* (Cas-OFFinder)	ATGAACATGgTGCACATCtTGG-NGG
Off-target 8: *LHFP* (Cas-OFFinder)	ATGAATGtTGCcAGCATCATGG-NGG
Off-target 9: *ANO4* (Cas-OFFinder)	ATGcATcATcCACATCATGG-NGG
Off-target 10: *IQCJ-SCHIP1* (Cas-OFFinder)	AgGAATGATGCAaAgCATGG-NGG
Off-target 11: *SEPT7* (Cas-OFFinder)	ATGAtTGATGCACATCATtt-NGG
Off-target 12: *TBCK* (Cas-OFFinder)	ATGAATGATGaACATCAacG-NGG
Off-target 13: *ELF2* (Cas-OFFinder)	ATGAAgGATGCACATCtgGG-NGG
Off-target 14: *RP11-122F24.1* (Cas-OFFinder)	ATGAATtATGtACATCATtG-NGG
Off-target 15: *CENPP* (Cas-OFFinder)	ATGAATGAaGCAgATCATGa-NGG
Off-target 16: *ARMC10* (crispr.mit.edu)	AgGCATGtTtCACATCATGG-NAG
Off-target 17: *GRM1* (crispr.mit.edu)	AatAATGATGtACATCtTGG-NAG
Off-target 18: *TTC21B-AS1* (crispr.mit.edu)	gTGAATGtgGCACATCATtG-NAG

Fig. S1, related to Fig. 1. CRISPR editing in human iPSCs to knock in *PIK3CA*^{H1047R}. A. Domain structure of the *PIK3CA* gene product p110α and schematic of the CRISPR/Cas9 targeting strategy showing the two homology-directed repair (HDR) templates used alone or in combination. **B.** Schematic of the sib-selection protocol used to isolate correctly targeted human iPSCs. In some cases, clonal lines were obtained without a need for a second round of subcloning (red arrow). **C.** Representative sequences of wild-type (*WT/WT*), *PIK3CA*$^{WT/H1047R}$ (*WT/H1047R*) and *PIK3CA*$^{H1047R/H1047R}$ (*H1047R/H1047R*) clones. All mutant clones are also homozygous for the two silent mutations introduced by the targeting strategy. The number of independent clones of each genotype generated is provided next to the chromatograms. **D.** A table of the 18 computationally predicted off-target sites that were examined in 2 wild-type, 3 *PIK3CA*$^{WT/H1047R}$ and 6 *PIK3CA*$^{H1047R/H1047R}$ clones. The following off-target sites were located in coding regions: 1, 2, 3, 16, 17 and 18. The sequencing failed for *SEPT7*, Off-target 11, due to poor primers. A heterozygous SNP (dbSNP ID: rs76736990) in *KALNR*, Off-target 4, was observed in all clones, suggesting that it was present in the original donor and not a consequence of CRISPR targeting. This was confirmed by examining the publicly available whole-genome sequencing data for the parental WTC11 cell line. The remaining off-target sites all had a wild-type sequence. **E.** Metaphase spreads confirming a normal 46XY karyotype in wild-type and *PIK3CA*^{H1047R} iPSC clones following gene editing. A total of 20 metaphases were examined in all cases except the parental clone before editing (11 metaphases analyzed).

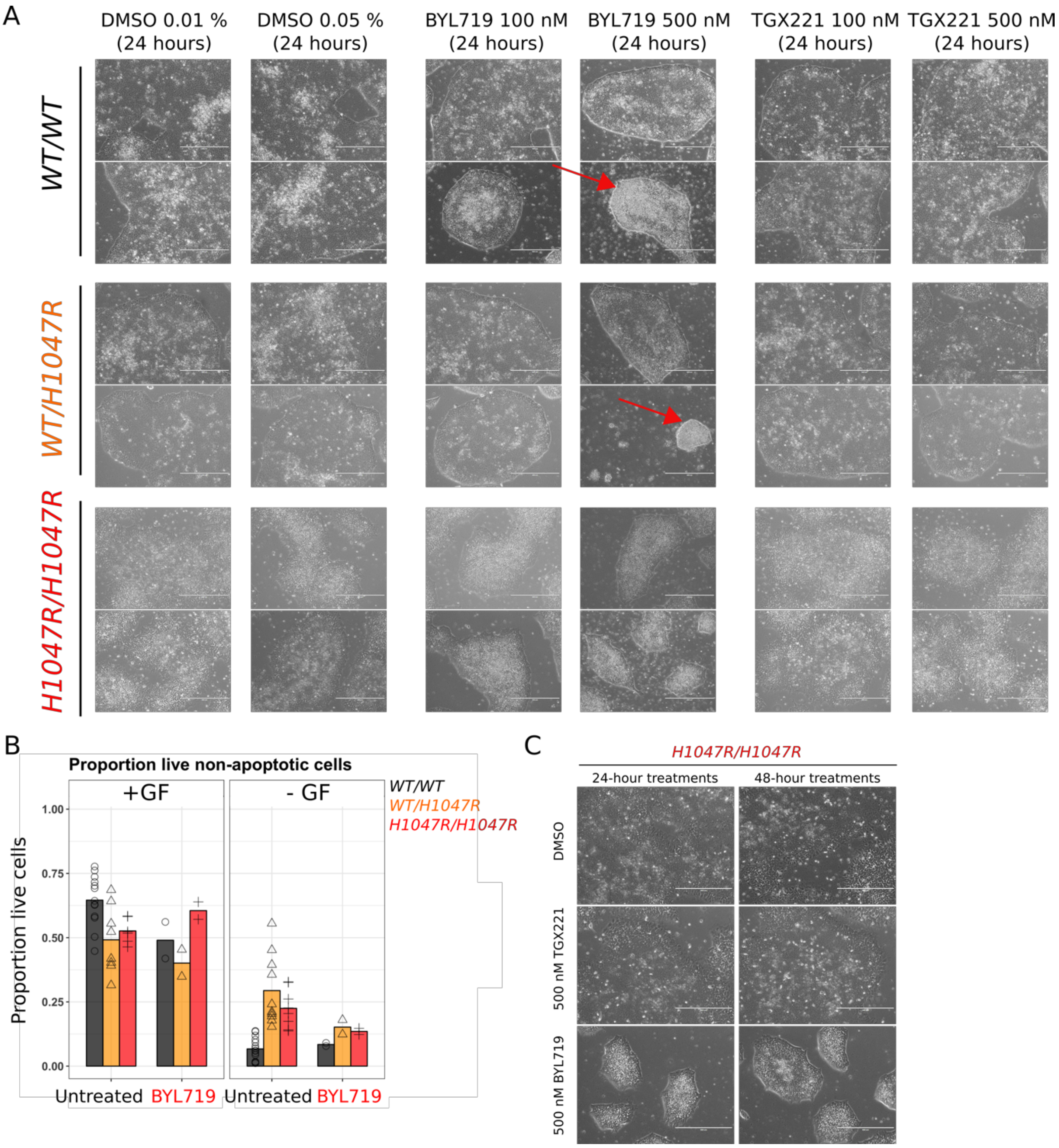

Fig. S2, related to Fig. 2. Drug response and survival phenotypes of wild-type and *PIK3CA*^{H1047R} iPSCs. **A**. The morphological appearance of the clones used to obtain the signaling data in Fig. 2C. Higher concentrations of BYL719 (500 nM) had cytotoxic effects in some of the wild-type (*WT/WT*) and *PIK3CA*$^{WT/H1047R}$ (*WT/H1047R*) clones (red arrows). This was not observed for *PIK3CA*$^{H1047R/H1047R}$ (*H1047R/H1047R*) clones. Scale bar: 400 µm. **B**. Cell viability at baseline and in response to 24 h growth factor (GF) depletion, +/- BYL719 (100 nM), was quantified by Annexin V staining of apoptotic cells combined with cell size discrimination of dead cells. Except for BYL719 treatments, which were performed in one experiment using technical duplicates of a single clone per genotype, data for the remaining treatments are from 3 independent experiments and at least 3 iPSC clones per genotype. Data points correspond to individual cell cultures, with bars representing the mean percentage of live cells for a given condition. **C**. The morphological appearance of *PIK3CA*$^{H1047R/H1047R}$ (*H1047R/H1047R*) clones treated with DMSO (control), 500 nM TGX221 (p110β inhibitor) or 500 nM BYL719 (p110α inhibitor). Representative of at least 3 experimental replicates and 6 *PIK3CA*$^{H1047R/H1047R}$ clones. Scale bar: 400 µm.

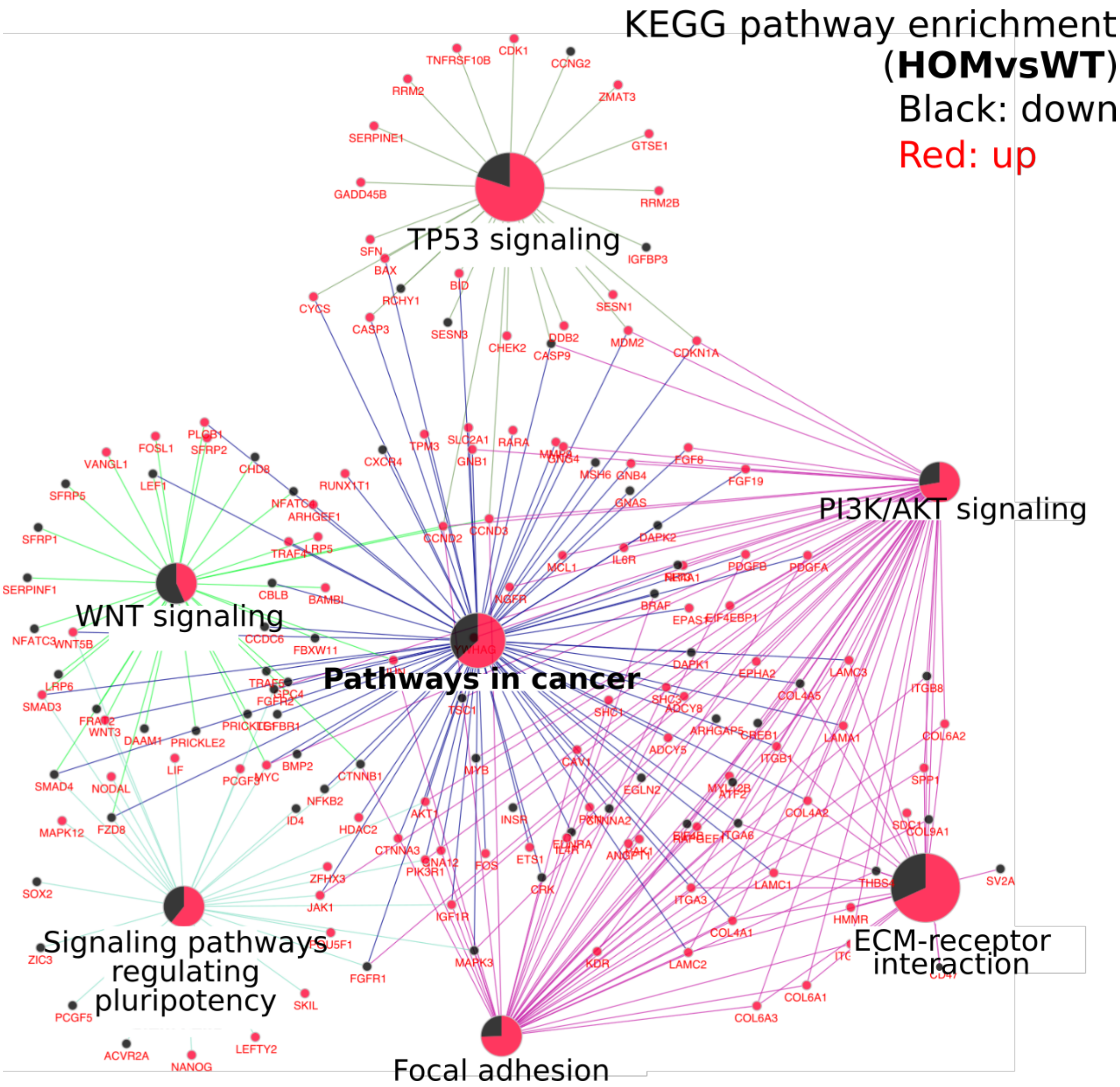

Fig. S3, related to Fig. 3. KEGG Pathway Enrichment Analysis. Undertaken using the Cytoscape plug-in ClueGO on the 1,914 differentially regulated transcripts in *PIK3CA*$^{H1047R/H1047R}$ iPSCs relative to wild-type controls (HOMvsWT). Pathways shown were significantly enriched with FDR = 0.05 (Benjamini-Hochberg). Differentially expressed genes belonging to enriched pathways are shown in red (upregulated) or black (downregulated). The proportions of upregulated and downregulated genes within a pathway are represented in central pie charts. ECM, extracellular matrix.

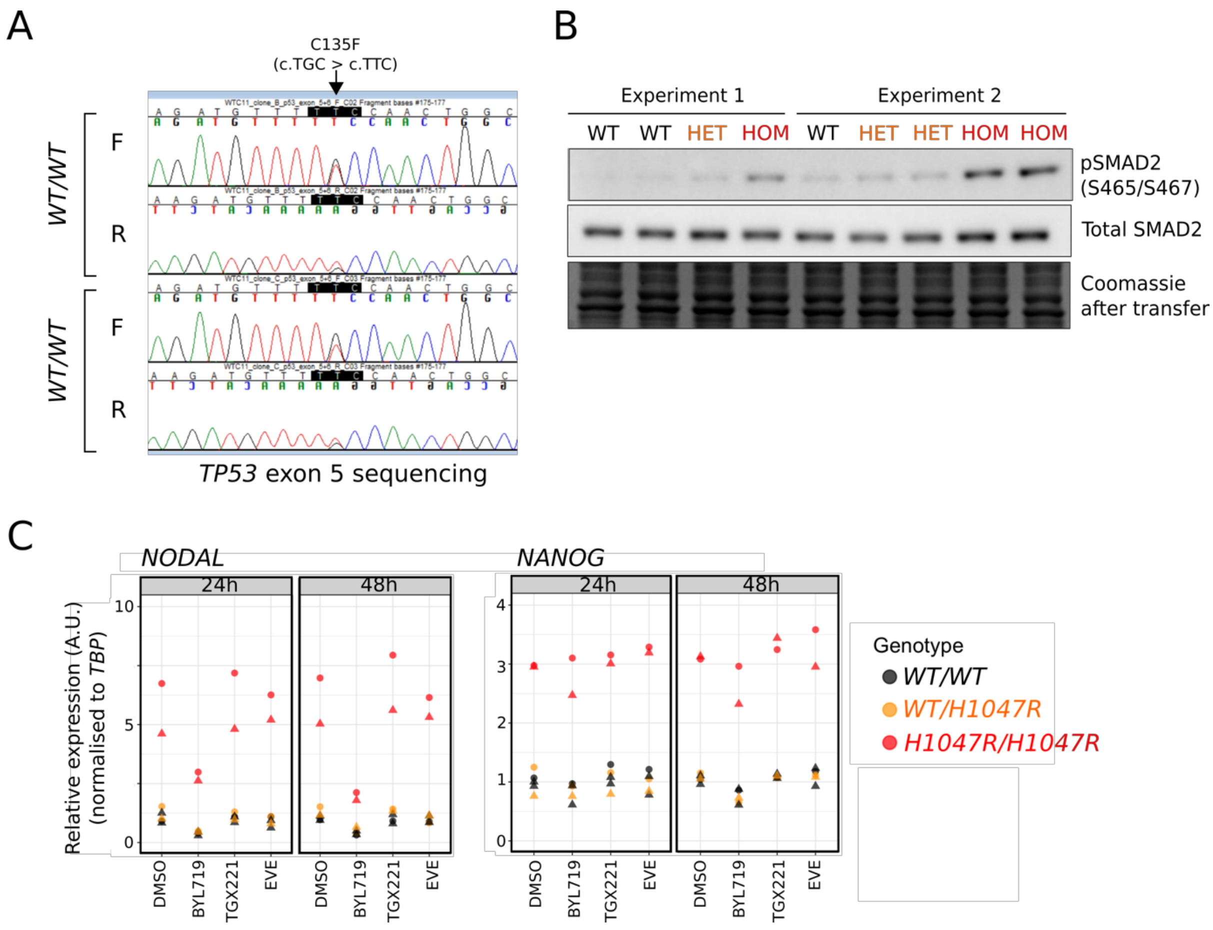

Fig. S4, related to Fig. 3. *TP53* sequencing, SMAD2 phosphorylation and regulation of *NODAL* and *NANOG* gene expression in wild-type and *PIK3CA*^{H1047R} iPSCs. **A.** Sanger sequencing traces of *TP53* exon 5 in two of the *PIK3CA* wild-type (*WT/WT*) cultures that had acquired a heterozygous *TP53*-C135F variant in culture. F, forward primer. R, reverse primer. **B**. Western blots for pSMAD2 (S465/S467) and total SMAD2 in wild-type (WT), *PIK3CA*$^{WT/H1047R}$ (HET) and *PIK3CA*$^{H1047R/H1047R}$ (HOM) iPSCs. Two experimental replicates are shown, conducted on 2 wild-type, 3 *PIK3CA*$^{WT/H1047R}$ and 2 *PIK3CA*$^{H1047R/H1047R}$ clones. **C**. RT-qPCR analysis of *NODAL* and *NANOG* expression in wild-type (*WT/WT*), *PIK3CA*$^{WT/H1047R}$ (*WT/H1047R*) and *PIK3CA*$^{H1047R/H1047R}$ (*H1047R/H1047R*) iPSCs following 24 h and 48 h treatments with either DMSO (control), 500 nM BYL719 (p110α inhibitor), 500 nM TGX221 (p110β inhibitor) or 5 nM everolimus (EVE; mTORC1 inhibitor). The data are from 2 independent experimental replicates, with 2 wild-type, 1 *PIK3CA*$^{WT/H1047R}$ and 2 *PIK3CA*$^{H1047R/H1047R}$ clone(s).

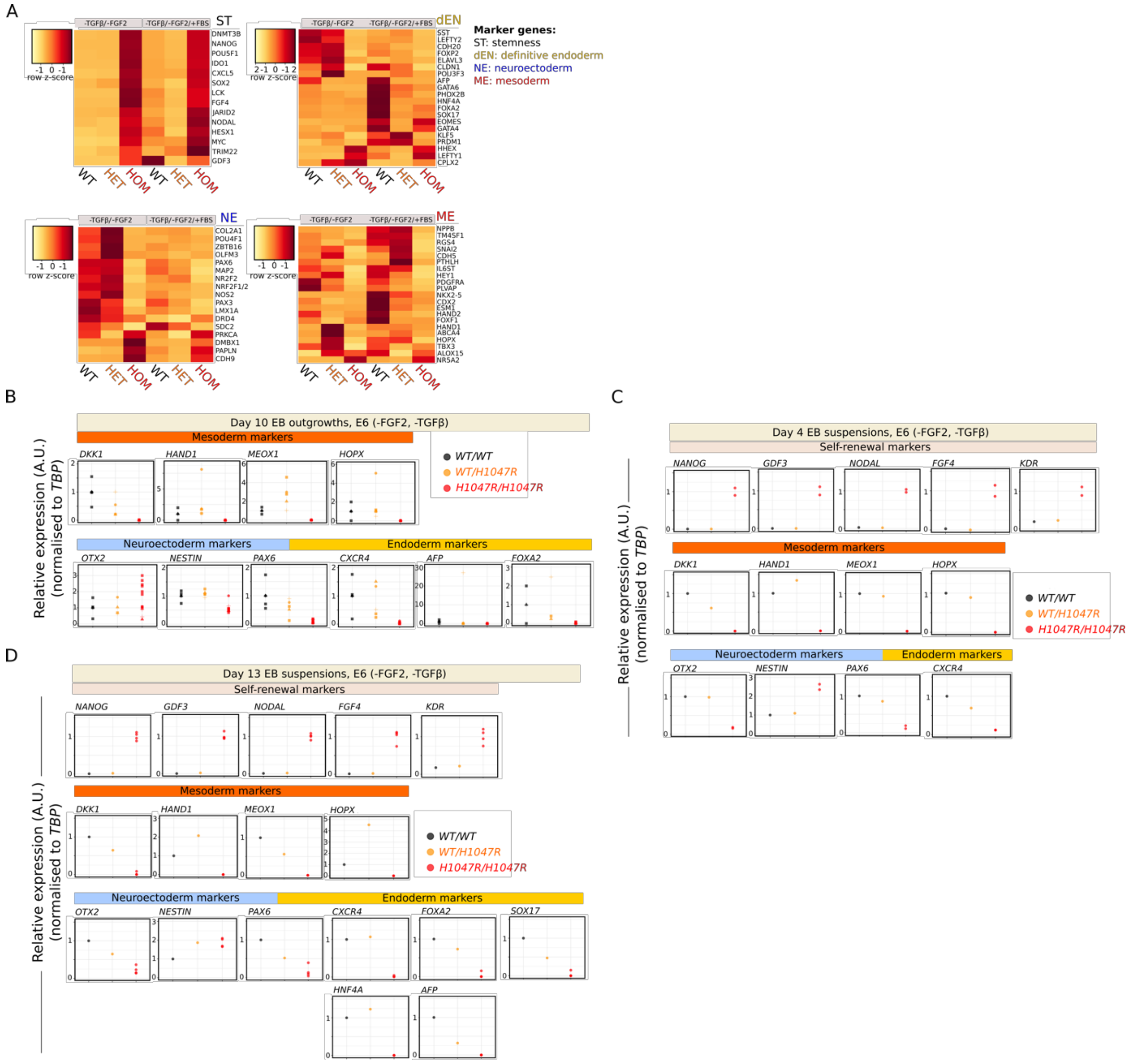

Fig. S5, related to Fig. 4. Lineage-specific gene expression in self-aggregating wild-type or *PIK3CA*^{H1047R} EBs under different conditions and at different time points. A. RT-qPCR scorecard-based profiling of lineage markers in wild-type (WT), *PIK3CA*$^{WT/H1047R}$ (HET) and *PIK3CA*$^{H1047R/H1047R}$ (HOM) EB outgrowths collected on day 10 of EB formation. The EBs were maintained in E6 throughout the protocol or in E6 supplemented with 10 % fetal bovine serum (FBS) from day 4 of EB formation, at the onset of adherent outgrowth formation. Gene heatmaps are shown across rows grouped according to lineage. Colors correspond to expression z-scores as indicated. The data are from a single experiment with one clone per genotype. **B**. As in Figure 5D but assessing additional lineage-specific markers as indicated. The data are from day 10 EB outgrowths maintained in E6 throughout the experiment. **C**. RT-qPCR analysis of the indicated lineage-specific markers in day 4 suspension EBs maintained in E6. The data are from a single experiment with one wild-type (*WT/WT*), one *PIK3CA*$^{WT/H1047R}$ (*WT/H1047R*) and 2 *PIK3CA*$^{H1047R/H1047R}$ (*H1047R/H1047R*) clones. **D.** RT-qPCR analysis of lineage-specific markers in day 13 suspension EBs maintained in E6. The data are from one experiment with one wild-type, one *PIK3CA*$^{WT/H1047R}$ and 4 *PIK3CA*$^{H1047R/H1047R}$ clones. Endoderm formation occurred at later stages of spontaneous EB differentiation, and the number of endoderm makers in each panel reflects the ability to detect these genes above background at each time point. All RT-qPCR expression values are normalized to *TBP* and scaled internally. Expression values are in arbitrary units (A.U.).

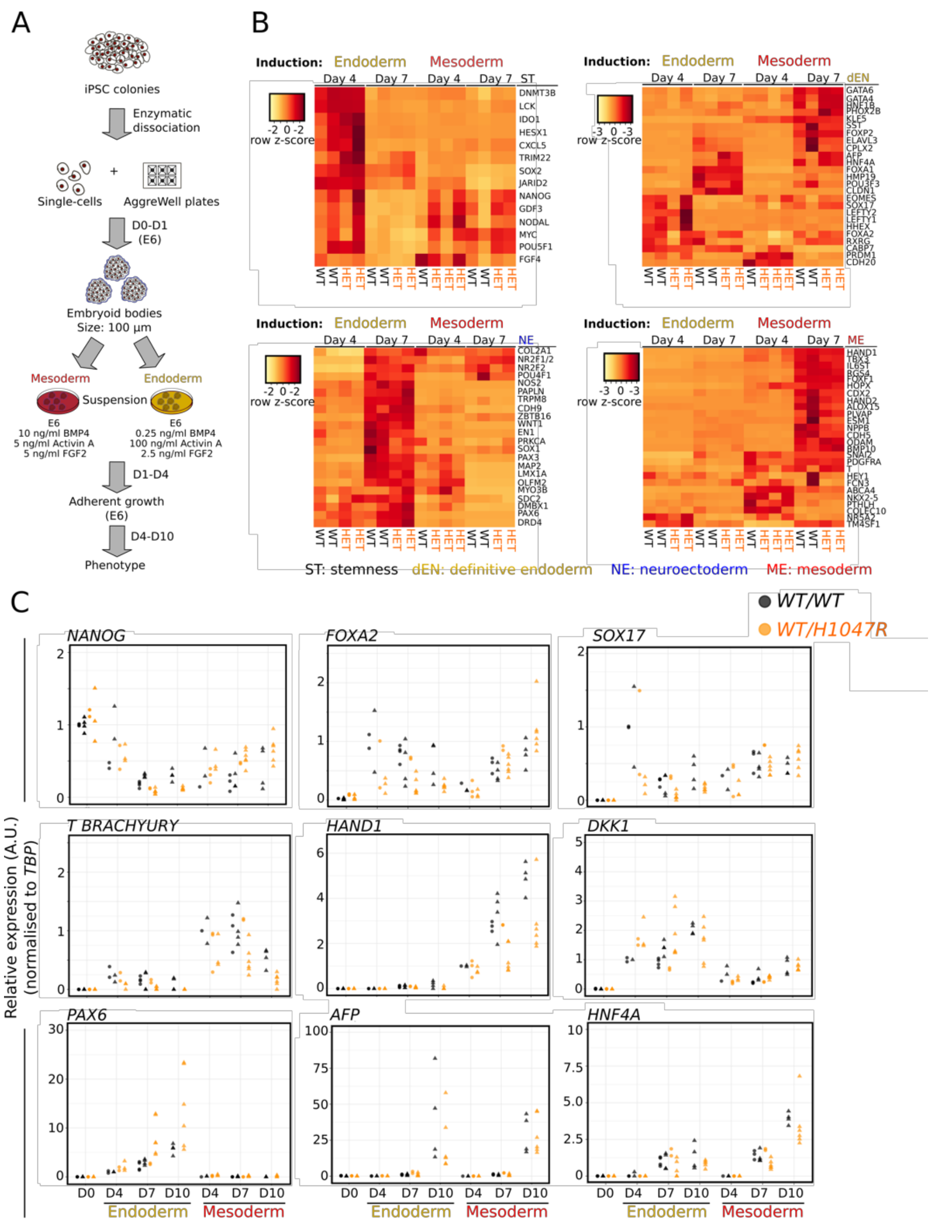

Fig. S6, related to Fig. 5. Lineage-specific gene expression in endo- or mesoderm-induced wild-type or *PIK3CA*$^{WT/H1047R}$ EBs. A. Schematic illustrating the AggreWell-based protocol for embryoid body (EB) culture with subsequent 3-day mesoderm or endoderm differentiation in suspension culture, followed by transfer to adherent conditions. D, day; E6, Essential 6 medium. **B.** RT-qPCR scorecard-based profiling of lineage markers in wild-type (WT) and *PIK3CA*$^{WT/H1047R}$ (HET) EBs following mesoderm or endoderm induction. Gene heatmaps are shown across rows and are grouped according to lineage. Colors correspond to expression z-scores as indicated. "Endoderm" and "Mesoderm" indicate induction conditions. Results are from a single experiment examining the following replicates: day 4 endoderm: 2 wild-type clones and 2 cultures of a single *PIK3CA*$^{WT/H1047R}$ clone; day 4 mesoderm: 1 wild-type clone and 3 *PIK3CA*$^{WT/H1047R}$ cultures from 2 clones; day 7 endoderm and mesoderm: 2 wild-type clones and 2 separate cultures of a single *PIK3CA*$^{WT/H1047R}$ clone. **C.** RT-qPCR assays of samples used in S6B plus additional replicates and time-points. Data shown for each timepoint represent 4-8 replicates from 2-4 clones. Gene expression values were normalized to *TBP* and scaled internally. The final values are in arbitrary units (A.U.). D, day.

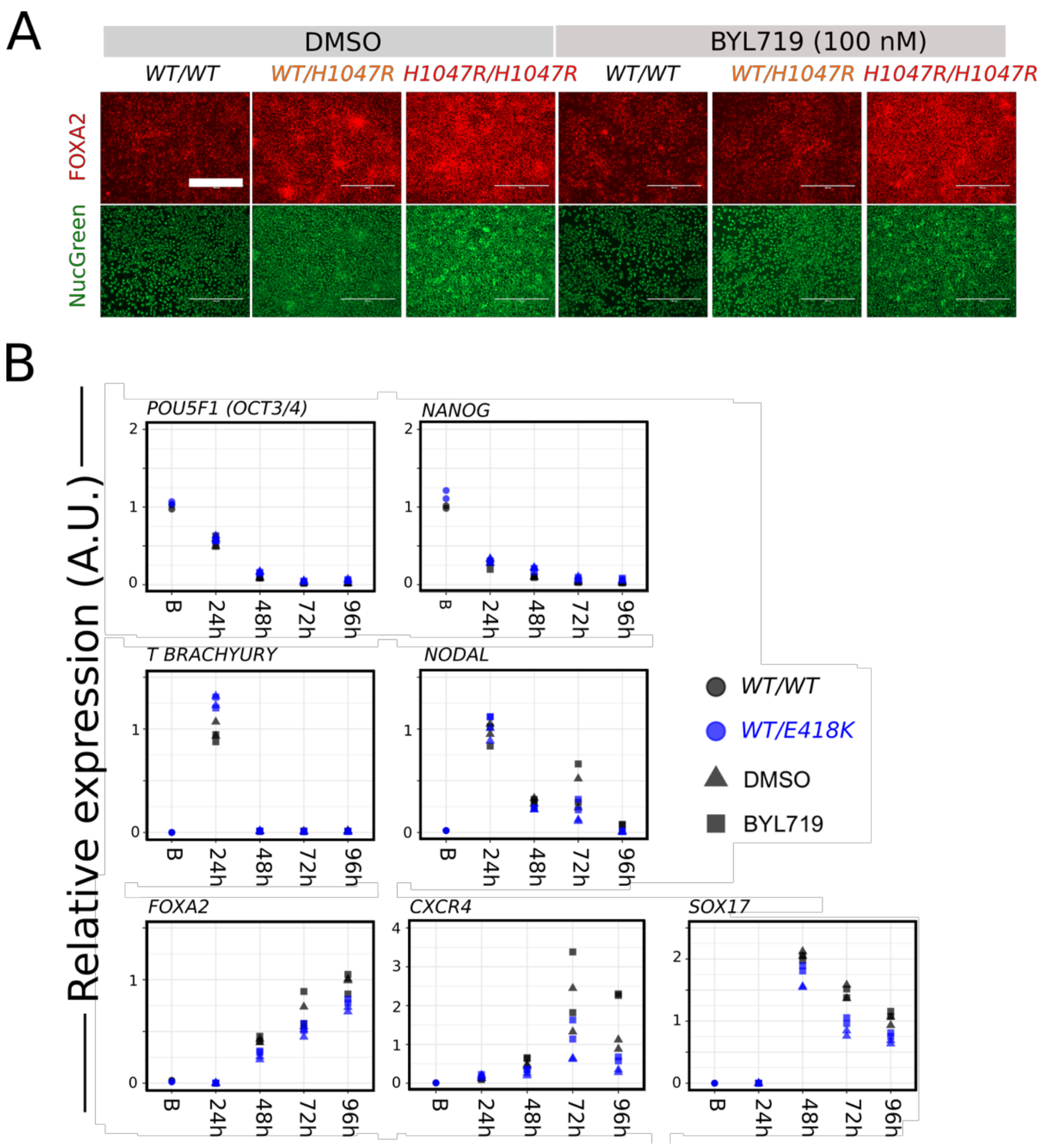

Fig. S7, related to Fig. 6. Gene expression analysis of monolayer-based endoderm differentiation of isogenic wild-type and *PIK3CA* mutant iPSCs. A. Immunofluorescence staining for FOXA2 in wild-type (*WT/WT*), *PIK3CA*$^{WT/H1047R}$ (*WT/H1047R*) and *PIK3CA*$^{H1047R/H1047R}$ (*H1047R/H1047R*) definitive endoderm cultures at the end of differentiation. NucGreen was used for nuclear visualization. Note the higher cell density in *PIK3CA*$^{H1047R/H1047R}$ cultures, attributable to improved survival following single-cell passaging. Scale bar: 400 µm. **B.** RT-qPCR gene expression analysis of lineage-specific markers throughout the course of monolayer-based definitive endoderm formation in genetically-matched wild-type (*WT/WT*) or *PIK3CA*$^{WT/E418K}$ (*WT/E418K*) iPSCs derived from a female PROS patient. The data are from a single experiment with one clone per genotype, with duplicate cultures of each clone. In both A and B, differentiations were performed in the presence of DMSO (control) or 100 nM BYL719.

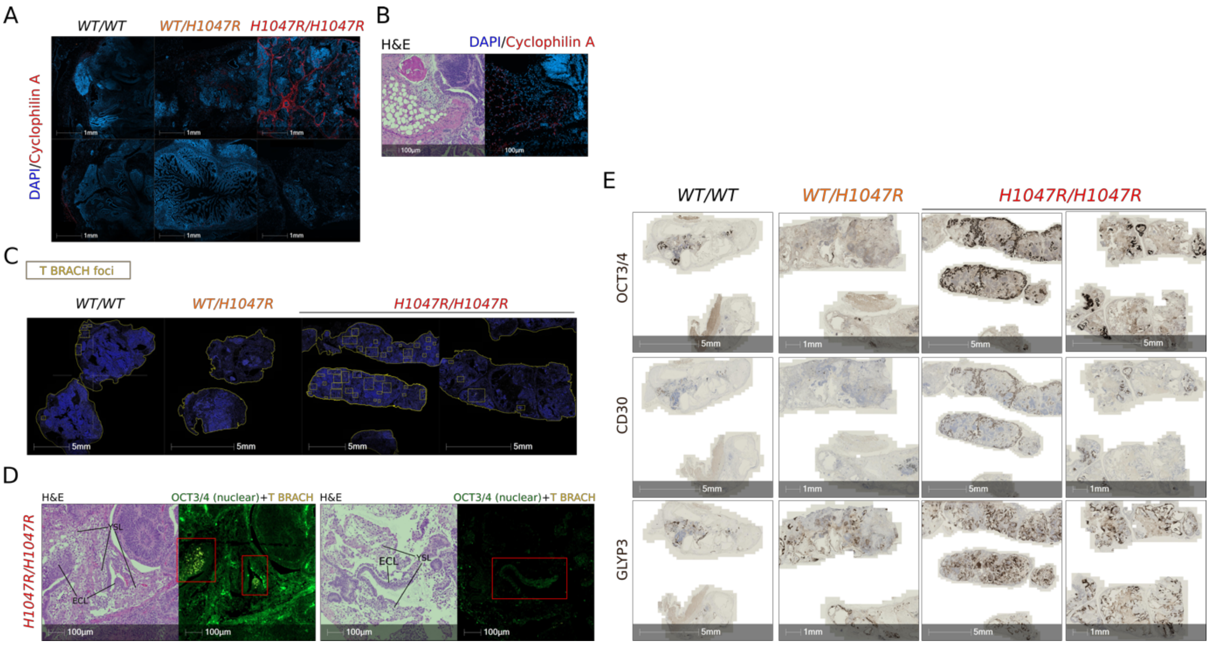

Fig. S8, related to Fig. 7. Characterization of xenograft tumors derived from wild-type or *PIK3CA*^{H1047R} iPSCs. A. Cyclophilin A-based detection of mouse host tissue in immunofluorescent-stained slides from human iPSC-derived tumor xenograft. DAPI was used for nuclear visualization. The immunofluorescence micrographs are representative of 4 WT (*WT/WT*) tumors from 3 different iPSC clones, 3 *PIK3CA*$^{WT/H1047R}$ (*WT/H1047R*) tumors from 3 different clones, and 2 *PIK3CA*$^{H1047R/H1047R}$ (*H1047R/H1047R*) tumors from 2 different clones. Scale bar: 1 mm. **B.** Example of matched hematoxylin and eosin (H&E) and DAPI/Cyclophilin A-stained slides from a wild-type tumor, demonstrating that the observed adipose tissue is of mouse origin. Representative of observations in the remaining tumors. **C**. Automated detection of T BRACHYURY (T BRACH)-positive cells in immunofluorescent stained slides from human iPSC-derived tumor xenografts. Areas with increased density of T BRACH-positive cells were marked manually as indicated by the yellow rectangles. Scale bar: 5 mm. **D**. Matched H&E and OCT3/4/T BRACHYURY immunofluorescent stained slides from the two *PIK3CA*$^{H1047R/H1047R}$ tumors. The OCT3/4 signal can be distinguished by its nuclear localization. Scale bar: 100 µm. ECL, embryonal carcinoma-like tissue; YSL, yolk sac-like tissue. **E.** Immunohistochemistry for OCT3/4 (embryonal carcinoma marker), CD30 (embryonal carcinoma marker) and GLYP3 (yolk sac tumor marker). Sample replication as in S8A. Scale bars are 1-5 mm as shown.

Table S1, Related to Fig. 7 and Fig. S8. Summary of the histopathological evaluation of wild-type and *PIK3CA*^{H1047R} tumor xenografts. The tumors were obtained from 5 wild-type (WT), 3 *PIK3CA*$^{WT/H1047R}$ (HET) and 2 *PIK3CA*$^{H1047R/H1047R}$ (HOM) iPSC cultures, using independent mutant clones and four wild-type clones. Six hematoxylin and eosin-stained slides with two different areas from each tumor were analyzed blindly by a human pathologist. [a] Presumed endoderm. [b] The quantification was performed subjectively by the pathologist, and the numbers are only approximate. (?) Sparse and therefore less certain appearance. In addition to the indicated tissues, all tumors also contained mature adipose tissue and blood vessels. However, mouse-specific Cyclophilin A-staining showed that these were not of human origin (see Fig. S8A and S8B).

	Endoderm			Mesoderm		Ectoderm			Other		Overall maturity
	GI epithelium	Respiratory epithelium	Immature unilayered tubular structures[a]	Cartilage	Bone	Pigmented epithelium [b]	Sebaceous-like glands	Neurones (ganglion cells)	Yolk sac-like elements	Necrosis [b]	
WT			+	+	+	10 %	+			< 5 %	++
WT			+		+	15 %				< 5 %	++/+++
WT			+	+	+	10 %	+	+		< 5 %	++
WT			+		+	< 5 %			Focal (?)	< 5 %	+/++
WT			+		+	< 5 %			Focal (?)	< 5 %	+/++
HET	+	+	+	+		< 5 %	+	+		< 5 %	+++
HET		+	+	+	Focal	10 %	+	+		< 5 %	++
HET		+	+			10 %		+		10 %	++
HOM			+		Focal				Focal	30 %	+
HOM			+						++	20 %	-/+

Table S2. Cell lines

Cell line	Source	Identifier
WTC11 male parental iPSC line	Coriell Institute	GM25256
WTC11-derived male wild-type iPSC line, post-CRISPR editing	This paper	N/A
WTC11-derived male *PIK3CA*$^{WT/H1047R}$ line	This paper (CRISPR knock-in)	N/A
WTC11-derived male *PIK3CA*$^{H1047R/H1047R}$ line	This paper (CRISPR knock-in)	N/A
M98-WT female iPSC line	This paper (reprogramming of patient-derived fibroblasts)	N/A
M98-E418K (*PIK3CA*$^{WT/E418K}$) female iPSC line	This paper (reprogramming of patient-derived fibroblasts)	N/A

Table S3. Nucleic acid sequences of sgRNA-Fb and the two HDR templates used to target *PIK3CA* exon 21. All sequences are provided in the 5’-to-3’ direction.

Component	Sequence
sgRNA-Fb	ATGAATGATGCACATCATGG
HDR001<br>(H1047R + silent mutations)	TAGCCTTAGATAAAACTGAGCAAGAGGCTT<br>TGGAGTATTTCATGAAACAAATGAACGACG<br>CACGTCATGGTGGCTGGACAACAAAAATGG<br>ATTGGATCTTCCACACAATTAAACAGCATG<br>CATTGAACTGAAAAGATAACTGAGAAAAG
HDR002<br>(“mock” – silent mutations only)	TAGCCTTAGATAAAACTGAGCAAGAGGCTT<br>TGGAGTATTTCATGAAACAAATGAACGACG<br>CACATCATGGTGGCTGGACAACAAAAATGG<br>ATTGGATCTTCCACACAATTAAACAGCATG<br>CATTGAACTGAAAAGATAACTGAGAAAAG

Table S4. Genotyping primers. F and R denote forward and reverse direction, respectively; all given in the 5'-to-3' direction. "-nest" in the ID indicates that the primer was used in nested PCR reactions, with "outer" and "inner" specifying that the primer was used in the first and second amplification, respectively. Primers used to sequence *PIK3CA* across codon 1047 required 56 °C during annealing. The annealing temperature for the remaining primers was 60 °C. RFLP, restriction fragment length polymorphism.

Primer ID	Sequence	Description
PR005	CAGCATGCCAATCTCTTCAT	Forward primer for sequencing of *PIK3CA* exon 21
PR006	ATGCTGTTCATGGATTGTGC	Reverse primer for sequencing of *PIK3CA* exon 21
PR009-F	FAM-TGATGCTTGGCTCTGGAATG	RFLP for *PIK3CA* exon 21; annealing T = 56 °C for 20 or 30 s. Use with PR010-R.
PR010-R	GGTCTTTGCCTGCTGAGAGT	RFLP for *PIK3CA* exon 21; annealing T = 56 °C for 20 or 30 s. Use with PR009-F.
Off-target-1F	TATGGCTCCTCTGATTGCACC	Off-target sequencing
Off-target-1R	CAGGCACCCATTTCTGGTAA	Off-target sequencing
Off-target-2F	GAGCCACGGACTTCCAGATAG	Off-target sequencing
Off-target-2R	CTAGCACAGGGCCACACAAAT	Off-target sequencing
Off-target-3F	GTGAAGTGAGGCGTGATGGG	Off-target sequencing
Off-target-3R	AGCAGAAGACCTCAACCATCC	Off-target sequencing
Off-target-4F-nest	CTACCTCCAAGCACATGGCA	Off-target sequencing
Off-target-4R-nest-inner	TGTGCCTCAATGTGCAAAGC	Off-target sequencing
Off-target-4R-nest-outer	TGAGCCCTTACCCTGTAGGG	Off-target sequencing
Off-target-5F-1	CAGAAATTTAGGGAAGGTGAATGG	Off-target sequencing
Off-target-5F-2	AGTTATCTGTTGTACAACATAGTGCC	Off-target sequencing
Off-target-5R-1	GATGTGTAAGGGAGAGCTGGTA	Off-target sequencing
Off-target-5R-2	CAGGATCAGATGGATTCACAACA	Off-target sequencing

Off-target-6F	GCCACACTCCACTGGTAGAC	Off-target sequencing
Off-target-6R	CAGTGTGCGTCTTCCCTTCT	Off-target sequencing
Off-target-7F	TTCTGGACCCCACCTCAGTA	Off-target sequencing
Off-target-7R	CCTTGAACATAGCCCCACCA	Off-target sequencing
Off-target-8F	TGGCTGGTAAGCACTCTCCT	Off-target sequencing
Off-target-8R	CCCCTGGCCTTTACGCTAAC	Off-target sequencing
Off-target-9F	TGCAGACTGAGAGTGCATGT	Off-target sequencing
Off-target-9R	GGCAGCTAGGGTTCTCAGTG	Off-target sequencing
Off-target-10F	ATTGTGGGACCACAGGAACA	Off-target sequencing
Off-target-10R	GGGCAAGGAAGCACTCTTGT	Off-target sequencing
Off-target-12F	CACATGACAGCTCATGGTGATG	Off-target sequencing
Off-target-12R	TGTGGAGGGCAACATACATCT	Off-target sequencing
Off-target-13F	CTGATGCTGCCCCTATACCA	Off-target sequencing
Off-target-13R	AAACTACCTGACACACTATAAGCA	Off-target sequencing
Off-target-14F	CCTTACACTGGCTGACCCTG	Off-target sequencing
Off-target-14R	GGGGCCACATCTTACTGCTT	Off-target sequencing
Off-target-15F	CTCAAAAGTCGCAGTGGCTC	Off-target sequencing
Off-target-15R	GTGACCAAATGGAAGCTGGC	Off-target sequencing
Off-target-16F	CTGGAAGAAGGGACGTCAGAG	Off-target sequencing
Off-target-16R-2	AGCAAATGGTACTGTCAAGTCC	Off-target sequencing
Off-target-17F	TATGCCTTCAAGACCCGCAA	Off-target sequencing

Off-target-17R	AAGCTTCCGCTTCTAGGCAAG	Off-target sequencing
Off-target-18F	CAACCCTCCCTGAGTACGTG	Off-target sequencing
Off-target-18R	TGTGGGTCATCGAGGGTCTG	Off-target sequencing
PIK3CAex21region-1F	TGCTGTGAAGGAAAATGGAAAGG	Tiled primer matrix, *PIK3CA* exon 21
PIK3CAex21region-2F	TACAACACAGTCCTGACTCTAGC	Tiled primer matrix, *PIK3CA* exon 21
PIK3CAex21region-3F	ACTGAGCAAGAGGCTTTGGA	Tiled primer matrix, *PIK3CA* exon 21
PIK3CAex21region-4F	CGCAAACAGGGTTTGATAGCAC	Tiled primer matrix, *PIK3CA* exon 21
PIK3CAex21region-5F	TGGGGTGGAAGGGACTCTTG	Tiled primer matrix, *PIK3CA* exon 21
PIK3CAex21region-6F	TTGCTCCAAACTGACCAAACT	Tiled primer matrix, *PIK3CA* exon 21
PIK3CAex21region-1R	AGTTTGGTCAGTTTGGAGCAA	Tiled primer matrix, *PIK3CA* exon 21
PIK3CAex21region-2R	CGGTCTTTGCCTGCTGAGA	Tiled primer matrix, *PIK3CA* exon 21
PIK3CAex21region-3R	CAGCCTTTGTTGTGTCCACATT	Tiled primer matrix, *PIK3CA* exon 21
PIK3CAex21region-4R	GACTGCTTCCAAAACTGCAGA	Tiled primer matrix, *PIK3CA* exon 21
PIK3CAex21region-5R-1	GTATTCAGTTCAATTGCAGAAGGAG	Tiled primer matrix, *PIK3CA* exon 21
PIK3CAex21region-5R-2	GCTGACCATGCTGCTATGAAC	Tiled primer matrix, *PIK3CA* exon 21
PIK3CAex21region-6R	GTGCTATCAAACCCTGTTTGCG	Tiled primer matrix, *PIK3CA* exon 21
TP53-exon2+3-F-nest-outer	GCTTGGGTTGTGGTGAAACA	*TP53* sequencing
TP53-exon2+3-F-nest-inner	GCAGCCATTCTTTTCCTGCTC	*TP53* sequencing
TP53-exon2+3-R-nest	GGTGAAAAGAGCAGTCAGAGGA	*TP53* sequencing
TP53-exon4-F-nest	AGAGACCTGTGGGAAGCGAA	*TP53* sequencing
	CAGGAAGCCAAAGGGTGAAGA	*TP53* sequencing

TP53-exon4-R-nest-outer		
TP53-exon4-R-nest-inner	GGATACGGCCAGGCATTGAA	*TP53* sequencing
TP53-exon5+6-F	GAGGTGTAGACGCCAACTCT	*TP53* sequencing
TP53-exon 5+6-R	CCACTGACAACCACCCTTAAC	*TP53* sequencing
TP53-exon7-F	CTCATCTTGGGCCTGTGTTATCT	*TP53* sequencing
TP53-exon7-R	GTGATGAGAGGTGGATGGGTAG	*TP53* sequencing
TP53-exon8+9-F	CTACCCATCCACCTCTCATCAC	*TP53* sequencing
TP53-exon8+9-R	ATGCCCCAATTGCAGGTAAAAC	*TP53* sequencing
TP53-exon10-F-nest	AATGCATGTTGCTTTTGTACCGTC	*TP53* sequencing
TP53-exon10-R-nest-outer	CCCTGGGTTTGGATGTTCTG	*TP53* sequencing
TP53-exon10-R-nest-inner	TGACCATGAAGGCAGGATGA	*TP53* sequencing
TP53-exon11-F-nest	AGCATTGGTCAGGGAAAAGG	*TP53* sequencing
TP53-exon11-R-nest-outer	AAGCAAGGGTTCAAAGACCCA	*TP53* sequencing
TP53-exon11-R-nest-inner	CAAAACCCAAAATGGCAGGGG	*TP53* sequencing

Table S5. SYBR Green qPCR primers. Bp, base pairs; Fwd, forward; Rev; reverse. Note that the lineage specification is only guiding as most genes are also expressed in other lineages; thus, multiple markers were assessed to determine lineage identity. Only a single accession ID is given due to space constraints, but several of the primers detect additional splice isoforms (of relevance for *TBP*, *POU5F1*, *NANOG*, *NODAL*, *FOXA2*, *CXCR4*, *HNF4A*, *PAX6, OTX2*).

**Target**	**Accession ID (RefSeq)**	**Fwd primer**	**Rev primer**	**Amplicon size (bp)**
POU5F1 (OCT3/4)	NM_002701.5	TGTACTCCTCGGTCCCTTTC	TCCAGGTTTTCTTTCCCTAGC	150
NANOG	NM_024865.3	CAGTCTGGACACTGGCTGAA	CTCGCTGATTAGGCTCCAAC	149
FGF4	NM_002007.2	CCAACAACTACAACGCCTACGA	CCCTTCTTGGTCTTCCCATTCT	82
GDF3	NM_020634.2	TTTCTCCCAGACCAAGGTTTC	TCCCTTTCTTTGATGGCAGA	107
NODAL	NM_018055.4	CAGTACAACGCCTATCGCTGT	TGCATGGTTGGTCGGATGAAA	75
KDR (VEGFR2)	NM_002253.2	GTGATCGGAAATGACACTGGAG	CATGTTGGTCACTAACAGAAGCA	124
T BRACHYURY	NM_003181.3	GCAAAAGCTTTCCTTGATGC	ATGAGGATTTGCAGGTGGAC	144
HAND1	NM_004821.2	TCCGCAGAAGGGTTAAACAG	CAAGGCTGAAAATGAGACGC	99
DKK1	NM_012242.2	ACCAGCTATCCAAATGCAG	TCACAGGGGAGTTCCATAAA	181
SOX17	NM_022454.3	CGCACGGAATTTGAACAGTA	CAGTAATATACCGCGGAGCTG	149
FOXA2	NM_021784.4	GGAGCAGCTACTATGCAGAGC	CGTGTTCATGCCGTTCATCC	83
CXCR4	NM_003467.2	GCCTTATCCTGCCTGGTATTGTC	GCGAAGAAAGCCAGGATGAGGAT	130
AFP	NM_001134.2	GCTTTGCTGAAGAGGGACAA	ACACCGAATGAAAGACTCGT	105
HNF4A	NM_001287182.1	ATCGCAGATGTGTGTGAGTC	GCAGAAAGCTGGGATGTACT	78
PAX6	NM_001604.5	AACGATAACATACCAAGCGTGT	GGTCTGCCCGTTCAACATC	120
OTX2	NM_021728.3	CAAAGTGAGACCTGCCAAAAAGA	TGGACAAGGGATCTGACAGTG	179
TBP	NM_003194.4	TAATCCCAAGCGGTTTGC	TAGCTGGAAAACCCAACTTCT	170

Table S6. Primary antibodies used for immunocytochemistry (ICC) and immunohistochemistry (IHC). CST, Cell Signaling Technology; mAb, monoclonal antibody; pAb, polyclonal antibody.

Target	Catalog # (clone if mAb)	Vendor	Species	Conjugate	Dilution	Application
TUBB3	801201 (TUJ1)	Biolegend	Mouse	No	1:500 (2 µg/ml)	ICC
T BRACHYURY	AF2085	R&D	Goat pAb	No	1:100 (2 µg/ml)	ICC/IHC
α -SMA (ACTA2)	A5228 (1A4)	Sigma	Mouse mAb	No	1:200 (10 µg/ml)	ICC
HAND1	AF3168	R&D	Goat pAb	No	1:100 (2 µg/ml)	ICC
SOX17	AF1924	R&D	Goat pAb	No	1:200 (1 µg/ml)	ICC
FOXA2 (hHNF3β)	AF2400	R& D	Goat pAb	No	1:50 (4 µg/ml)	ICC
AFP	sc-8399 (C3)	Santa Cruz	Mouse mAb	No	1:100 (2 µg/ml)	ICC
AFP	14-9499-82 (1E8)	Thermo Fisher Scientific	Mouse mAb	No	1:100 (5 µg/ml)	IHC
OCT3/4	sc-5279 (C-10)	Santa Cruz	Mouse mAb	No	1:100 (2 µg/ml)	ICC/IHC
TRA-1-60	sc-21705	Santa Cruz	Mouse mAb	No	1:100 (2 µg/ml)	ICC
TRA-1-60	14-8863-82	Thermo Fisher Scientific	Mouse mAb	No	1:100 (5 µg/ml)	ICC
NANOG	AF1997	R&D	Goat pAb	No	1:100 (2 µg/ml)	ICC
NANOG	PA1-097	Thermo Fisher Scientific	Rabbit pAb	No	1:100 (10 µg/ml)	ICC
Cyclophilin A	51418 (D2Y4M)	CST	Rabbit mAb	No	1:100 (1.3 µg/ml)	IHC
Mouse IgG isotype control	I5381	Sigma	Mouse	No	1:1975 (2 µg/ml); 1:395 (10 µg/ml)	ICC
Mouse IgG isotype control	37355	Abcam	Mouse	No	1:2500 (2 µg/ml); 1:1000 (5 µg/ml); 1:500 (10 µg/ml)	IHC
Rabbit IgG isotype control	10500C	Thermo Fisher Scientific	Rabbit	No	1:1500 (2 µg/ml); 1:300 (10 µg/ml)	ICC
Goat IgG isotype control	ab37373	Abcam	Goat	No	1:1500 (2 µg/ml)	ICC/IHC

Table S7. Primary and secondary antibodies used for automated immunohistochemistry on the Leica BOND-III.

Primary Antibody	Catalogue #	Vendor	Species	Dilution	BOND-III antigen retrieval method	Secondary HRP-conjugated antibody
CD30	PA0790	Leica	Mouse	Ready to use	H1 (30)	Leica Bond Polymer Refine Detection Kit
GLYP3	261M-96	Cell Marque	Mouse	1:150	H2 (30)	Leica Bond Polymer Refine Detection Kit
OCT3/4	PA0934	Leica	Mouse	Ready to use	H2 (30)	Leica Bond Polymer Refine Detection Kit

Table S8. Primary antibodies used for Western blotting. CST, Cell Signaling Technology; kDa, kilodalton; mAb, monoclonal antibody; pAb, polyclonal antibody.

Target	Catalog # (clone if mAb)	Vendor	Species	Molecular weight (kDa)	Dilution	Application
FGFR1	9740 (XP D8E4)	CST	Rabbit mAb	92, 120, 145	1:1,000	Western
p110α	4249 (C73F8)	CST	Rabbit mAb	110	1:1,000	Western
p110β	3011 (C33D4)	CST	Rabbit mAb	110	1:1,000	Western
pAKT S473	9271	CST	Rabbit pAb	60	1:1,000	Western
AKT	9272	CST	Rabbit pAb	60	1:1,000	Western
pERK1/2 T202/Y204, T185/Y187	9106 (E10)	CST	Mouse mAb	42,44	1:1,000	Western
pERK1/2 T202/Y204, T185/Y187	4377 (197G2)	CST	Rabbit mAb	42,44	1:1,000	Western
pERK1/2 T202/Y204, T185/Y187	4370 (D13.14.4E)	CST	Rabbit mAb	42,44	1:1,000<br>1:2,000	Western
ERK1/2	4695 (137F5)	CST	Rabbit mAb	42,44	1:1,000	Western
pSMAD2 S465/S467	3108 (138D4)	CST	Rabbit mAb	60	1:1,000	Western
SMAD2	3122 (86F7)	CST	Rabbit mAb	60	1:1,000	Western

Table S9. Other materials. ICC, immunocytochemistry; IHC, immunohistochemistry; Molbio, molecular biology; WB, Western blotting.

Item	Catalogue #	Vendor	Application
10 % formaldehyde, Ultrapure, EM-grade, methanol-free,	4018	PolySciences	Imaging
100 ml DMSO, sterile-filtered	D2650	Sigma	Cells
2.5 % R.T.U. Horse Serum Blocking (IHC)	S2012	Vector Laboratories	Imaging
2X SYBR Green PCR Master Mix	4309155	Thermo Fisher Scientific	Molbio
2X TaqMan Universal PCR Master Mix	4318157	Thermo Fisher Scientific	Molbio
Activin A for endoderm/mesoderm EB studies	In-house	Cambridge LRM	Cells
Advanced RPMI 1640	12633-012	Thermo Fisher Scientific	Cells
Agarose (ultrapure)	16500	Sigma	Molbio
Agencourt CleanSEQ-Dye Terminator Removal	A29151	Beckman Coulter	Molbio
AggreWell 400, 24-well	34411	Stem Cell Technologies	Cells
AggreWell rinsing solution	7010	Stem Cell Technologies	Cells
Annexin V-Cy5 Apoptosis Staining/Detection Kit	ab14150	Abcam	Imaging
Basic FGF2 for endoderm/mesoderm EB studies	In-house	Cambridge LRM	Cells
BigDye Terminator v3.1 Cycle Sequencing Kit	4337455	Thermo Fisher Scientific	Molbio
Bioanalyzer DNA 12000 Kit	5067-1508	Agilent	MolBio
Bioanalyzer RNA 6000 Nano Kit	5067-1511	Agilent	Molbio
BmgBI RE	R0628L	NEB	Molbio
BMP4 for endoderm/mesoderm EB studies	In-house	Cambridge LRM	Cells
BSA-100g (for blocking)	A7906-100g	Sigma	Protein
BYL719	B9700-1mg	Cambridge Bioscience	Cells
CHIR99021	04-0004	Stemgent	Cells
cOmplete ULTRA Tablets, Mini, EasyPack, PhosStop	4906845001	Sigma	Protein
cOmplete, EDTA-free Protease Inhibitor Cocktail (tablets)	5056489001	Sigma	Protein
CytoPainter Phalloidin-iFluor 555 Reagent	ab176756	Abcam	Imaging
DAKO antigen retrieval solution pH = 6 (10X)	S2369	DAKO	Imaging
DAKO antigen retrieval solution pH = 9 (10X)	S2367	DAKO	Imaging
DAKO wash buffer 10X	S3006	DAKO	Imaging
DC Protein Assay Reagent A	5000113	Biorad	Protein
DC Protein Assay Reagent B	5000114	Biorad	Protein
DC Protein Assay Reagent S	5000115	Biorad	Protein
Direct-zol RNA Miniprep Kit	R2051	ZymoResearch	Molbio
DMEM/F12 with L-Glutamine, HEPES 500 ml	11330-032	Thermo Fisher Scientific	Cells

DMEM/F12 without L-Glutamine, HEPES 500 ml	D6421	Sigma	Cells
EndoFree Plasmid Maxi Kit (10)	12362	Qiagen	Molbio
Essential 6 Medium	A1516401	Thermo Fisher Scientific	Cells
Essential 8 Flex Medium Kit	A2858501	Thermo Fisher Scientific	Cells
Everolimus	HY-10218	MedChem Express	Cells
Exonuclease I	M0293L	NEB	Molbio
EZ Blue (Coomassie reagent)	G1041	Sigma	Protein
FACS tubes with cell strainer	352235	Corning	Cells/imaging
Fetal Bovine Serum (FBS)	SV30180.03	HyClone	Cells
Fluoroshield with DAPI	F6057	Sigma	Imaging
Fluoroshield without DAPI	F6192	Sigma	Imaging
Geltrex LDEV Free hESC Quality 5 ml	A1413302	Thermo Fisher Scientific	Cells
GeneScan-600 LIZ Size Standard	4366589	Applied Biosystems	Molbio
GlutaMAX	35050061	Thermo Fisher Scientific	Cells
Glycerol	15514-011	Sigma	Various
GoTaq MasterMix (2X)	M7122	Promega	Molbio
HEPES Buffer	H0887	Sigma	Protein
HiDi Formamide 25 ml	4211320	Thermo Fisher Scientific	Molbio
High-Capacity cDNA Reverse Transcription Kit	4368814	Thermo Fisher Scientific	Molbio
HiSpeed Plasmid Maxi Kit	12362	Qiagen	Molbio
Hoechst (Bisbenzimide H 33342 for fluorescence)	14533-100mg	Sigma	Imaging
Horse Serum Blocking (ICC)	30-0702	PAN-Biotech	Imaging
iBlot transfer stacks regular	IB301001	Thermo Fisher Scientific	Protein
Immobilon Western Chemiluminescent HRP Substrate	WBKLS0500	Merck Millipore	Protein
Ingenio Cuvettes 50 pk	MIR50121	Cambridge Bioscience	Nucleofection
KaryoMAX Colcemid Solution in PBS	15212012	Thermo Fisher Scientific	Cells
Leica Bond Epitope Retrieval Solution 1	AR9961	Leica	Imaging
Leica Bond Epitope Retrieval Solution 2	AR9640	Leica	Imaging
Leica Bond Polymer Refine Detection Kit	DS9800	Leica	Imaging
Matrigel, hESC-qualified	354277	Corning	Cells
mFreSR freezing reagent 50 ml	5855	Stem Cell Technologies	Cells
MinElute PCR Purification Kit 50 rxn	28004	Qiagen	Molbio
Nuclear Select FX Labelong kit	S33025	Thermo Fisher Scientific	Imaging
Nunclon Sphera 60 mm dish (21.5 cm^2)	174944	Thermo Fisher Scientific	Cells
NuPAGE 4-12% BT midi 12+2 well PAGE gels	WG14001BOX	Thermo Fisher Scientific	Protein

NuPAGE Antioxidant	NP0005	Thermo Fisher Scientific	Protein
NuPAGE LDS Sample Buffer (4X)	NP0007	Thermo Fisher Scientific	Protein
NuPAGE MOPS SDS Running Buffer 20X	NP0001	Thermo Fisher Scientific	Protein
NuPAGE Sample Reducing Agent (10X)	NP0009	Thermo Fisher Scientific	Protein
OneShot Stbl3 Chemically Competent Cells	C7373-03	Thermo Fisher Scientific	Molbio
PBS (Dulbecco's without calcium chloride and magnesium chloride)	D8537	Sigma	Cells
Phusion High-Fidelity DNA Polymerase	M0530L	NEB	Molbio
Precision Plus Dual Color Protein Standard	161-0374	Biorad	Protein
Prolong Gold Antifade reagent	P36930	Thermo Fisher Scientific	Imaging
PSC Cryopreservation Kit	A2644601	Thermo Fisher Scientific	Cells
Q5 High-Fidelity DNA Polymerase	M0491L	NEB	Molbio
QIAamp DNA Micro Kit (50)	56304	Qiagen	Molbio
QIAzol Lysis Reagent	79306	Qiagen	Molbio
QuickExtract DNA Extraction Solution	QE09050	Cambridge Bioscience	Molbio
Ready Probes Cell Viability Imaging Kit, Blue/Green	R37609	Thermo Fisher Scientific	Imaging
Recombinant human EGF 100 µg	AF-100-15	PeproTech	Cells
Recombinant human FGF2-basic (for GF stimulations)	100-18B	PeproTech	Cells
Recombinant human IGFI 100 µg	100-11	PeproTech	Cells
ReLeSR 100 ml	5872	Stem Cell Technologies	Cells
RevitaCell Suppplement 5 ml	A2644501	Thermo Fisher Scientific	Cells
RLT Buffer	79216	Qiagen	Molbio
RNeasy Mini Kit	74104	Qiagen	Molbio
SAP (Shrimp Alkaline Phosphatase)	GEE70092Z	Sigma	Molbio
Secondary antibody (ICC+IHC), Donkey anti-Goat IgG (H+L) Cross-Adsorbed Secondary Antibody, Alexa Fluor 647	A21447	Thermo Fisher Scientific	Imaging
Secondary antibody (ICC+IHC), Goat anti-Mouse IgG (H+L) Highly Cross-Adsorbed Secondary Antibody, Alexa Fluor 488	A11029	Thermo Fisher Scientific	Imaging
Secondary antibody (ICC), Goat anti-Rabbit IgG (H+L) Highly Cross-Adsorbed Secondary Antibody, Alexa Fluor 594	A11037	Thermo Fisher Scientific	Imaging
Secondary antibody (IHC), Goat anti-Rabbit IgG (H+L) Highly Cross-Adsorbed Secondary Antibody, Alexa Fluor 546	A110235	Thermo Fisher Scientific	Imaging

Secondary antibody (WB), Goat anti-Rabbit IgG HRP-linked Antibody	7074S	CST	Protein
Secondary antibody (WB), Horse anti-Mouse IgG HRP-linked Antibody	7076S	CST	Protein
StemPro Accutase	A110501	Thermo Fisher Scientific	Cells
SuperScript IV Fist Strand Synthesis System	18091050	Thermo Fisher Scientific	Molbio
TaqMan® hPSC Scorecard™ Panel, 384-well	A15870	Thermo Fisher Scientific	Molbio
TeSR-E8 Medium Kit	5940	Stem Cell Technologies	Cells
TGX221	S1169	SelleckChem	Cells
TrueSeq mRNA library kit	20020594	Illumina	Molbio
TruSeq RNA Single Indexes Set A	20020492	Illumina	Molbio
TrypLe Express Enzyme (1X), phenol red	12605010	Thermo Fisher Scientific	Cells
Wizard Plus SV Miniprep Kit	A1330	Promega	Molbio
β-mercaptoethanol	M7522	Sigma	Protein
μ-Dish 35 mm, low	80136	Ibidi	Imaging
μ-Slide 4 Well	80426	Ibidi	Imaging

References

1. Bae S, Park J, Kim JS (2014) Cas-OFFinder: A fast and versatile algorithm that searches for potential off-target sites of Cas9 RNA-guided endonucleases. *Bioinformatics*. doi:10.1093/bioinformatics/btu048.
2. Kosicki M, Tomberg K, Bradley A (2018) Repair of double-strand breaks induced by CRISPR-Cas9 leads to large deletions and complex rearrangements. *Nat Biotechnol* 36(8):765–771.
3. Diekmann U, Lenzen S, Naujok O (2015) A reliable and efficient protocol for human pluripotent stem cell differentiation into the definitive endoderm based on dispersed single cells. *Stem Cells Dev* 24(2):190–204.
4. Larionov A, Krause A, Miller W (2005) A standard curve based method for relative real time PCR data processing. *BMC Bioinformatics* 6:62.
5. Krämer A, Green J, Pollard J, Tugendreich S (2014) Causal analysis approaches in ingenuity pathway analysis. *Bioinformatics* 30(4):523–530.
6. McGranahan N, et al. (2015) Clonal status of actionable driver events and the timing of mutational processes in cancer evolution. *Sci Transl Med* 7(283):1–12.
7. Rosner M, Hengstschläger M (2018) An antibody-based approach for the in situ evaluation of mouse contribution in human stem cell-derived xenografts. *Protoc Exch*:1–35.
8. Liu P, Hwang JTG (2007) Quick calculation for sample size while controlling false discovery rate with application to microarray analysis. *Bioinformatics* 23(6):739–746.
9. Edgar R (2002) Gene Expression Omnibus: NCBI gene expression and hybridization array data repository. *Nucleic Acids Res* 30(1):207–210.
